# Synthesis and Characterization Superabsorbent Polymers Made of Starch, Acrylic Acid, Acrylamide, Poly(Vinyl Alcohol), 2-Hydroxyethyl Methacrylate, 2-Acrylamido-2-methylpropane Sulfonic Acid

**DOI:** 10.3390/ijms22094325

**Published:** 2021-04-21

**Authors:** Elżbieta Czarnecka, Jacek Nowaczyk

**Affiliations:** 1 Faculty of Chemistry, Nicolaus Copernicus University in Toruń, 7 Gagarina Street, 87-100 Toruń, Poland; jacek.nowaczyk@umk.pl; 2 Plastica Sp. z o.o., Frydrychowo 55, 87-410 Kowalewo Pomorskie, Poland

**Keywords:** semi-interpenetrating network hydrogel, biomaterial, superabsorbent polymer, hydrogel, graft copolymerization

## Abstract

Three polymers with excellent absorption properties were synthesized by graft polymerization: soluble starch-g-poly(acrylic acid-co-2-hydroxyethyl methacrylate), poly(vinyl alcohol)/potato starch-g-poly(acrylic acid-co-acrylamide), poly(vinyl alcohol)/potato starch-g-poly(acrylic acid-co-acrylamide-co-2-acrylamido-2-methylpropane sulfonic acid). Ammonium persulfate and potassium persulfate were used as initiators, while *N,N*′-methylenebisacrylamide was used as the crosslinking agent. The molecular structure of potato and soluble starch grafted by synthetic polymers was characterized by means of Fourier Transform Infrared Spectroscopy (FTIR). The morphology of the resulting materials was studied using a scanning electron microscope (SEM). Thermal stability was tested by thermogravimetric measurements. The absorption properties of the obtained biopolymers were tested in deionized water, sodium chroma solutions of various concentrations and in buffer solutions of various pH.

## 1. Introduction

In 1976, the United States Department of Agriculture introduced polymers with water absorbency called superabsorbent polymers (SAPs) [1], which have found wide application in many industries including: gardening, agriculture, cosmetics, controlled drug release, hygiene products, and wastewater treatment [2,3]. These extraordinary polymer materials consist of weakly cross-linked chains having hydrophilic moieties, allowing them to retain water up to thousands of times their own weight [4]. Most of the SAPs industrially available are produced from synthetic polymers, mostly by the copolymerization of vinyl monomers specifically including acrylic or methacrylic acid derivatives [3]. Therefore, the market of SAPs is dominated by petroleum-based synthetic polymers having a long decomposition time, which inflicts a negative impact on the environment [5].

The vast majority of SAPs produced recently are used in disposable products and subsequently dumped in landfills or utilized by incineration; in both cases they are not safe for ecosystems [6]. Nowadays, many people care about their own physical and mental health, thus they become increasingly aware of environmental protection and the diversified development of natural resources. Consequently, the industry tends to deliver more ecological and biodegradable products in order to fulfill consumers’ expectations. The increasing interest in the polysaccharides as an excellent renewable raw material fits this trend. Polysaccharides have more advantages; they are cheap, nontoxic biodegradable, and easy to modify. However, in the field of SAPs polysaccharides did not meet the requirements in terms of absorption and mechanical properties [7]. For this reason, a lot of research effort had been employed both in academic and industrial institutions to solve the problem [8].

There has been a lot of research on the chemical modification and cross-linking of cellulose. However, starch modification was also tested with various synthetic monomers, e.g., acrylic acid [9,10,11,12,13,14,15], acrylamide [16,17,18], methacrylamide [19,20], acrylonitrile [21], vinyl alcohol [22], 2-acrylamido-2-methylpropanesulfonic acid [23,24], styrene [25], vinylimidazole [26].

Starch is a very popular source material for the production of biodegradable goods and for years was an important subject of research [27]. It is the second most abundant natural carbohydrate with many advantages including low price, nontoxicity, ease of chemical modification and processability. Obtained from renewable sources such as potato, corn, wheat and rice and capable of being processed in water-based solvents, starch is definitely a green material considered as a potential alternative to some petroleum-based polymers [28]. Morphologically, starch consists of nearly spherical granules of diameter between 1 and 150 µm. Chemically, it is composed of two similar polysaccharides—amylose and amylopectinboth—built of glucose rings connected by α-glycoside bonds. The former forms mostly linear chains composed of α-D-(1–4) glucan units. In contrast, amylopectin is a highly branched version of α-D-(1–4) glucan chains linked together with α-D-(1–6) glycosidic linkages at branch points. Starch is completely soluble in water above 130° and in alkali solutions at lower temperatures. The rapid cooling of concentrated starch solutions results in formation of hydrogel, while slow cooling of dilute solutions can lead to crystallization of amylose components. Long amylose chains as well as long amylopectin branches can crystallize, adopting helical conformation with hydrophilic core and helix twist formed of about six glucose units. Strong hydrogen bonding between glucose OH groups from neighboring chains or chain segments prevents the polymer from easy swelling and dissolution. Dry starch granules absorb little water, retaining their initial crystallinity and hydrogen bonding structure. It requires significantly elevated temperature to loosen the compact basic structure and let the solvent inside granules. Therefore, starch gelation occurs in high temperatures when the interchain forces are weakened enough to allow water molecules to penetrate the interchain space. As a result, nonmodified starch cannot be used as an efficient water absorbent.

Despite the abovementioned drawbacks, starch still remains in the hot-spot of scientific interest as a promising biodegradable absorbent polymer. The main reason is the ease of the chemical modification of starch, allowing researchers to tailor its properties according to specific needs. In the case of superabsorbents, the polymer is expected to easily absorb high amounts of water and water-based solutions at room temperature. Other requirements are the preservation of a shape—in other words, a polymer cannot dissolve in the solvent releasing fluids. To change starch into such a material it is necessary to modify its chemical structure to facilitate the penetration of the interchain space by the water molecules. Polysaccharides are modified in this direction by grafting hydrophobic side-chains to the carbohydrate main chain. The technique has been extensively studied in the case of cellulose [29]; however, recently one can also find numerous studies of starch modification [30]. Another approach employs the formation of an interpenetrating network (IPN) of starch and nonionic or ionic hydrogel [31]. Recently semi-interpenetrating network hydrogels (SIPNH) are often studied as a combination of both previous approaches [32].

In the present study, we investigated the synthesis and usefulness of the modification of starch in order to obtain a semi-interpenetrating network hydrogel utilizing graft copolymerization of acrylic acid (AA), 2-hydroxyethyl methacrylate (HEMA), acrylamide (AM), and/or 2-acrylamido-2-methylpropane sulfonic acid (AMPS) for starch backbones. Additionally, we studied the effect of poly(vinyl alcohol) (PVA) addition. The polymerization of acrylic acid [33] and acrylamide [34] leads to formation of hydrophilic chains, improving the water absorption properties of the system [35]. Linear poly(vinyl alcohol) possesses a flexible structure, excellent permeability of fluids, and low interfacial tension [36,37] due to the presence of the hydroxyl pendant groups. PVA has many advantages, such as including good swelling capacity and nontoxicity; however, it forms unstable hydrogels because their structure is maintained by weak hydrogen bonds [38]. Nevertheless, PVA was found to be a good additive to other, more stable hydrogels, improving their sorption effectiveness. Typically, adding PVA to other water swollen polymers led to the formation of IPN hydrogel.

**Figure 1:**
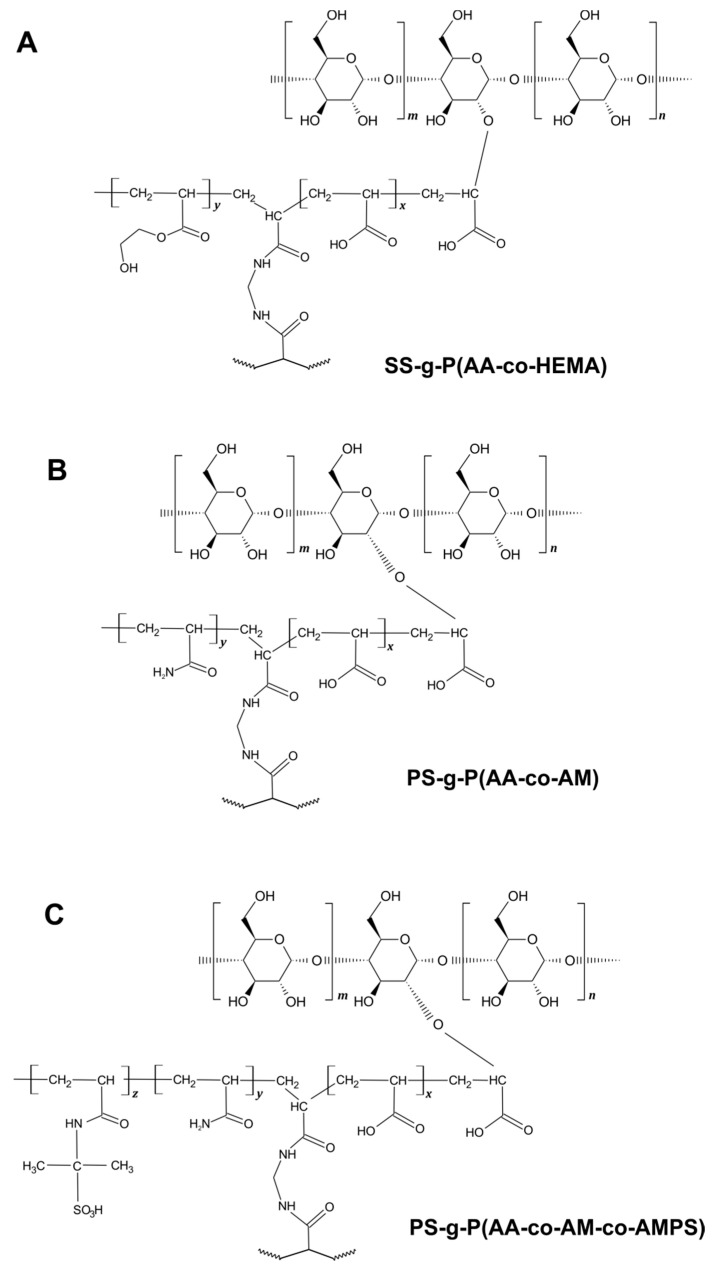
Schematic representation of synthesized grafted starch counterparts of superabsorbent polymers: soluble starch-g-poly(acrylic acid-co-2-hydroxyethylmethacrylate) SS-g-P(AA-co-HEMA) (**A**), potato starch-g-poly(acrylic acid-co-acrylamide) PS-g-P(AA-co-AM) (**B**), and poly(vinyl alcohol)/potato starch-g-poly(acrylic acid-co-acrylamide-co-2-acrylamido-2-methylpropane sulfonic acid) PS-g-P(AA-co-AM-co-AMPS) (**C**).

In the present study, we have achieved the synthesis and characterization of the three starch-based superabsorbents schematically depicted in Figure 1. The schemes presented represent possible reaction products based on the literature. One is a crosslinked graft copolymer of starch modified with AA and HEMA (Figure 1A). The second comonomer was used to increase the hydrophilicity of the resulting network [20]. The other two schemes are of SIPNH type superabsorbent materials with graft-modified starch as the main component and PVA as a linear hydrophilic counterpart. Similar studies have already been published but mostly in the context of the synthesis route. In our study, we have undertaken the effort to investigate and compare three different gel systems. Although the methods of starch modification with acrylic monomers are known in the literature, the products usually suffer from poor sorption abilities and water retention. Addressing this issue, we have chosen an original combination of monomers and compared three different structural modes. For the sake of comparison, the simple and widely published starch-g-poly(acrylic acid-co-2-hydroxyethyl methacrylate) was synthesized and subjected to similar tests. In our study SS and PS starch were modified by the combination of the following monomers: AA and AM (Figure 1B); AA, AM and AMPS in the second case (Figure 1C). The AMPS was used to introduce a large number of ionizable groups –COO^−^ and –SO_3_^−^, which can increase the swelling capacity [39]. The presence of different comonomers in the polymer chain provides different hydrophobicity, leading to a pH-sensitive material [40].

## 2. Experimental Methods

### 2.1. Materials

In the recent work we have used polymer substrates such as potato starch (PS) ACS reagent grade (Sigma Aldrich, Poznań, Poland), soluble starch (SS) pure (Sigma Aldrich, Poznań, Poland), and poly(vinyl alcohol) (PVA) pure (Sigma Aldrich, Poznań, Poland). We have used acrylic acid (AA) pure (Sigma Aldrich, Poznań, Poland); 2-hydroxyethyl methacrylate (HEMA) pure (Sigma Aldrich, Poznań, Poland), and 2-acrylamido-2-methylpropane sulfonic acid (AMPS) pure (Sigma Aldrich, Poznań, Poland) as grafting monomers. Potassium persulfate (KPS) analytical grade (Sigma Aldrich, Poznań, Poland) was used as the reaction initiator. *N,N*′–methylenebisacrylamide (MBA) pure (Sigma Aldrich, Poznań, Poland) was applied as crosslinking reagent. Besides, we have also used other auxiliary reagents such as sodium hydroxide (NaOH) (Sigma Aldrich, Poznań, Poland); nitrogen gas (N_2_) technical grade; ethanol 96 vol% (Bioetanol AEG Ltd., Chełmża, Poland). All these compounds were used as obtained. Necessary solutions were prepared using deionized water.

### 2.2. Infrared Spectroscopy

The identification of reaction products was conducted by means of Fourier transform infrared spectroscopy (FTIR). Dry pot material was analyzed at room temperature in horizontal attenuated total reflectance (ATR) mode with a diamond crystal. FTIR spectra in the range between 4000 and 400 cm^−1^ were collected using a Bruker Vertex 70 V spectrometer (Bruker Optoc GmbH, Ettlingen, Germany). For each sample for 16 scans with a resolution of 4 cm^−1^ was recorded and averaged using built-in machine routine. The resulting spectra have been normalized analyzed using OPUS 7.5 software (Bruker Optoc GmbH, Ettlingen, Germany).

### 2.3. Thermal Analysis

Thermal properties of materials were analyzed using a Simultaneous TGA-DTA thermal-analyzer type SDT 2960 Simultaneous TGA-DTA from TA Instruments, Champaign, IL, USA) at temperatures ranging from 20 °C to 1000 °C. For all studied samples of ca. 2–4 mg a heating rate of 10 °C/min was applied and tests were carried out under atmospheric air. The device was specified with a dynamic temperature precision within the limits of ±0.5 °C and a calorimetric accuracy/precision of ±2% (based on metal standards). Recorded thermograms were analyzed using TA Universal Analysis Software. The analysis was employed to investigate a pristine polymer as well as a polymer after 10 cycles of swelling/drying.

### 2.4. Scanning Electron Microscopy

Surface topography and size of superabsorbent particles were tested using a scanning electron microscope manufactured by LEO Electron Microscopy Ltd. Cambridge, UK, model 1430 VP. The sample was tested as dry granules (not milled) with gold coating. The apparatus was working in SE mode under the following conditions: accelerating voltage 10 kV, working distance about 11 mm (exact WD values are given in the figures). The surface of the granules was analyzed at three sites. The test samples were dried immediately before the analysis under vacuum at the temperature of 50.0 °C ± 0.1 °C for 24 h. Scanning electron microscopy was used to determine the shape, size, and morphology of superabsorbent polymers.

### 2.5. Preparation of Soluble Starch-G-Poly(Acrylic Acid-Co-2-Hydroxyethylmethacrylate)

**Figure 2:**
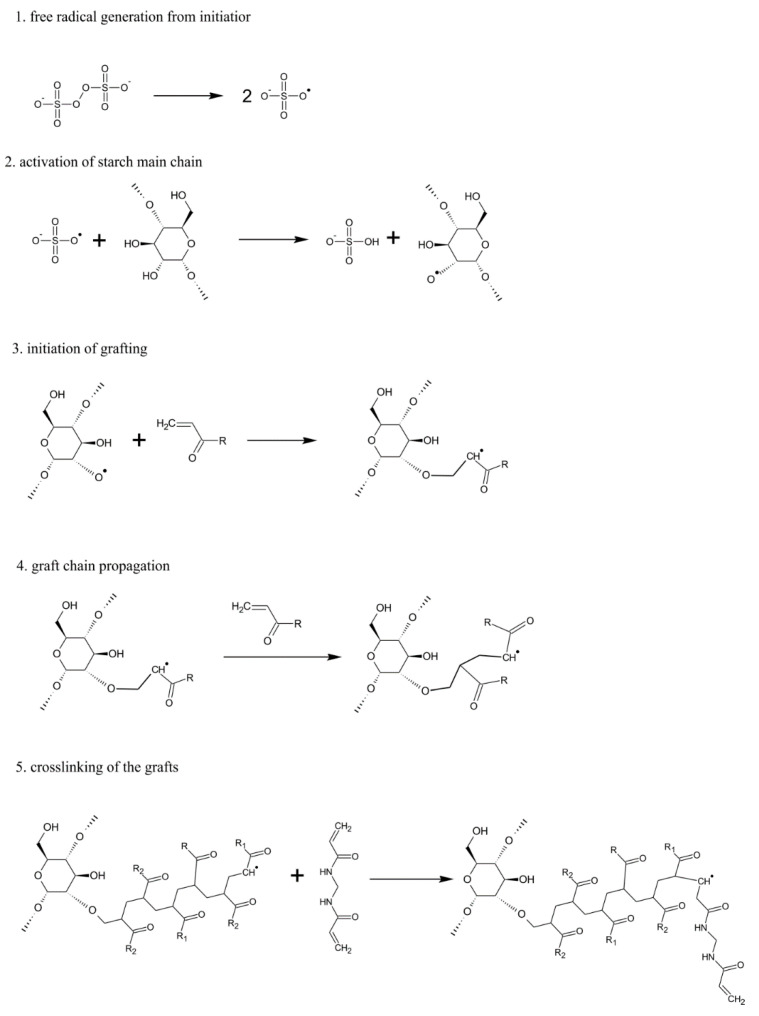
Possible mechanism of the grafting/crosslinking reaction of starch, acrylate monomers and MBA crosslinker.

We mixed 2.0149 g of soluble starch and 35 mL of deionized water in a 250 mL round bottomed flask. The system was heated to 80 °C by the heating mantle and stirred with a mechanical stirrer at a speed of 195 rpm for 30 min. Once the starch was gelatinized, the flask with its contents was transferred to a water bath and a nitrogen inlet was mounted. The temperature was adjusted to 60 °C, and then 0.092 g/mol KPS solution was added. After 15 min, a mixture of monomers containing 1.512 g AA and 1.514 g HEMA was added to the continuously stirred mixture. After another 15 min crosslinker was introduced to the mixture (0.067 g/mol MBA). The flask content was stirred for 3 h under a nitrogen atmosphere using a mechanical stirrer at the temperature of 60 °C. Afterwards the system was cooled to room temperature and the mixture pH was adjusted to 9.5 with solution of NaHCO_3_ 0.5 g/mol and 10 wt% NaOH solution. The resulting gel was washed thoroughly with ethanol, which was filtered off after 4 h. The product was dried in a vacuum oven for 48 h at 50 °C. After drying, the obtained product was ground in a laboratory mill (IKA A-10) and stored in a desiccator, away from moisture, light, and heat. The synthesis mechanism scheme is given in Figure 2, showing subsequent stages of the process. This mechanism is similar in all cases studied here.

### 2.6. Preparation of Poly(Vinyl Alcohol)/Potato Starch-G-Poly(Acrylic Acid-Co-Acrylamide)

**Figure 3:**
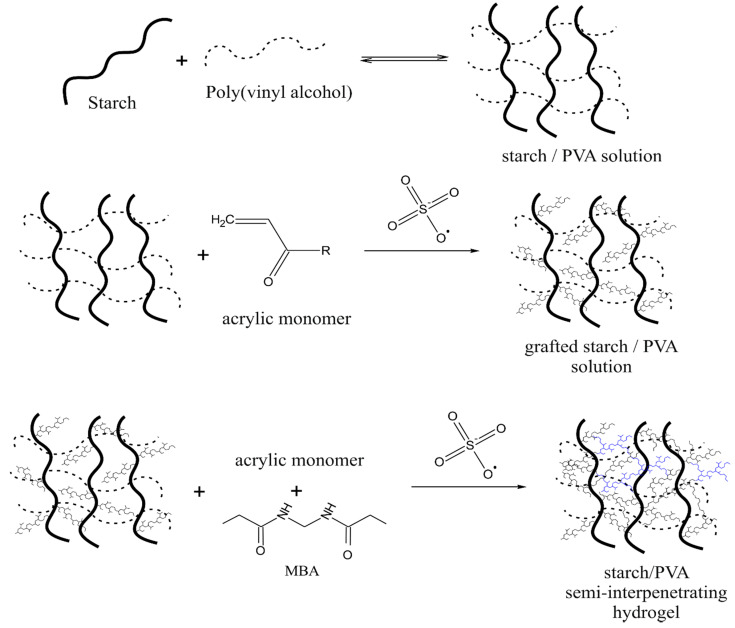
Possible mechanism of a semi-interpenetrating network hydrogel formation from crosslinked starch and linear PVA.

A three-necked round-bottom flask 250 mL was filled with 3.020 g of potato starch, 18 mL of distilled water and 0.9 mL, of 40 wt% NaOH solution. The flask was placed in a water bath at the temperature of 40 °C under a nitrogen atmosphere and stirred with a mechanical stirrer. When the starch was gelatinized, to the mixture was added: 10 mL of 2.817 g/mol AM solution and 17.992 g AA neutralized with NaOH solution. After 30 min, the temperature was raised to 50 °C and 0.015 g of KPS was added as initiator and 15 min later 20 mL of PVA aqueous solution (2006 g PVA) was added. The mixture was stirred for 30 min and a solution of crosslinking agent (0.068 g/mol MBA) was introduced. Afterwards the mixture turned into a thick gel and was stirred for 4 h using a mechanical stirrer. The obtained product was removed from the flask and cut into pieces 5 × 5 mm. The pieces were dried for 120 h at ambient temperature, and then transferred to a vacuum oven at 50 °C for 24 h. After drying, it was ground into small pieces in an IKA A-10 basic laboratory mill. The mechanism of semi-interpenetrating network hydrogel formation according to this synthesis is schematically depicted in Figure 3.

### 2.7. Preparation of Poly(Vinyl Alcohol)/Potato Starch-G-Poly(Acrylic Acid-Co-Acrylamide-Co-2-Acrylamido-2-Methylpropane Sulfonic Acid)

Firstly, a solution consisting of 1.0631 g PVA dissolved in 50 mL of deionized water was poured into a three-necked, round bottomed flask. Then, 1.0764 g of potato starch, pregelatinized in 50 mL of deionized water was added and the mixture was stirred using a mechanical stirrer under nitrogen atmosphere at 60 °C. After about 30 min, 0.343 g/mol KPS solution was added and during 15 min, the temperature was decreased to 40 °C. Then, we poured 30 mL of monomer solution containing 3.339 g A, 3.600 g AM and 3.600 g AMPS into the mixture and stirred it. Finally, a solution of crosslinker 1.503 g/mol of MBA was added. The content of the flask was alkalinized to pH 11 using NaOH solution. The mixture was kept for 3 h at 70 °C to complete the reaction. The product obtained was washed with 80 vol% ethanol and dried in a vacuum oven at 50 °C for 48 h. After drying, it was ground into small pieces in an IKA A-10 basic laboratory mill.

### 2.8. Swelling Characteristics

The swelling characteristics including properties such as degree of swelling (Q_t_), equilibrium swelling (Q_eq_) and swelling rate, have been determined for starch and all new SAP materials. To determine the equilibrium swelling, expressing maximal mass of water per 1 g of superabsorbent, about 0.1000 g of dried polymer sample was dispersed in double distilled water to swell for 24 h. After filtration, the extracted gel was reweighed and Q_eq_ was calculated using following formula:

(1) $$Q_{\mathrm{eq}}=\frac{w_{s}-w_{d}}{w_{d}}$$

where w_d_ and w_s_ are the weights [g] of the dry sample and water swollen sample, respectively.

The degree of swelling was determined in the similar way but the sample was removed from the solvent (water, NaCl soln., or buffer) at certain time dried form the excess of surface water weighted and then returned to the solvent. The degree of swelling (also referred to as water absorbency) was determined using following formula:

(2) $$Q_{t}=\frac{w_{t}-w_{d}}{w_{d}}$$

where w_t_ is the weights [g] of the swollen sample at a given time. The pH-dependent swelling experiments were carried out by immersing about 0.1000 g of dried superabsorbent in solutions with a defined pH at 25 °C for 24 h. Defined pH buffer solutions were prepared from 0.1 M HCl and 0.1 M NaOH solution (controlled by a pH meter by ChemLand, model 7011-01. Weight of swollen samples was measured after surface drying with filter paper. We used1 wt%, 2 wt%, 4 wt% and 8 wt%. NaCl solutions were t test the ionic strength on the samples.

### 2.9. Swelling Dynamics

The swelling properties of superabsorbent hydrogels, such as water absorption and fluid retention, were studied according to common methods described in the literature [41]. The absorbency rate of the studied absorbents was measured for ground particle samples of approximately 0.100 ± 0.001 g. Dry absorbent powder was inserted into weighted teabags and then dipped in 250 mL of distilled water. The kinetics of swelling is complex process, which is usually described mathematically using empirical models. Description of this process is often narrowed to investigation of initial swelling rate, i.e., when the swelling is significantly below 60%. Initial swelling rate is determined from the formula derived from Voigt viscoelasticity model combining a spring with dashpot. The equation allows us to manage a fast transition from a high initial rate toward a very slow rate near the end of the process [12]. Using this approach, one comes to following equation:

(3) $$Q_{t}=Q_{\mathrm{eq}}\left(1-\exp\left(-\frac{t}{\tau }\right)\right)$$

where τ is so-called “rate parameter”, which in the original Voigt model is referred to as the “retardation time” and as such determines the influence of the dashpot.

There are a number of factors that may influence the swelling characteristics and kinetics in particular. Among the most notable are pH, temperature, solvent properties, and structural parameters of polymer network. In this work we have found that the Q_t_ = *f*(t) plot can be described using simple power law equation in the following form:

(4) $$Q_{t}=A\cdot \exp\left(-b\cdot t^{-\frac{1}{2}}\right)$$

where A and b are empirical coefficients. However, detailed analysis revealed that these parameters could be given specific phenomenological explanation. Thus, A is an estimate of the equilibrium swelling (Q_eq_) while b corresponds with the diffusion rate of the solvent in polymer interchain spaces. The model accuracy is expressed by R^2^ and Fit Standard Error (FSE) provided in tables together with equation coefficients.

## 3. Results and Discussion

### 3.1. Analysis of the Synthesis Mechanism

Various types of monomers, such as AA, AM, AMPS, and HEMA, have been used in a homogeneous medium to graft starch backbone. The reaction was initiated by KPS and the graft chains were crosslinked using MBA. The reaction mechanism is visualized in Figure 2 and Figure 3, indicating the most important reaction stages and the phenomenology of the IPN formation. Initially, the thermal dissociation of KPS results in the formation of sulfate radical anion, which is assumed to extract the hydrogen atom from the hydroxyl group of starch’s glucose unit at position two or three, which forms a radical. The monomer molecules close to the macroradical sites scavenge the unpaired electron attaching to polysaccharide backbone. This mechanism of initiation is fundamentally different from the oxidative radical initiation by Ce^4+^ ions, which form radical sites by the cleavage of the C2–C3 bond of the glucose unit. The influence of initiation on the structure and properties of the resulting hydrogel was discussed in our previous paper [12]. Since the monomer attached to glucose unit has a radical site it starts the chain growth process according to propagation reaction of chain polymerization. The monomers employed in the graft chain’s formation possess ionophore groups, thus, the resulting network is rich in hydrophilic, ionizable groups such as anionic –COO^−^, –SO_3_^−^, and cationic–^+^NH_2_–improving aqueous liquids’ absorption. The use of inert monomers (acrylamide, 2-hydroxyethyl methacrylate) may facilitate the control of swelling, and sensitivity to pH of the environment. The system PVA/PS-g-P(AA-co-AM) is formed by partially interpenetrating polymer networks schematically depicted in Figure 3. The linear PVA polymer chains penetrate the 3D network of amylase and amylopectin, which undergo grafting. Grafted and crosslinked starch introduce chemically crosslinked polymer network that is additionally physically crosslinked by hydrogen bonding with PVA. This kind of interaction between the chains improves the stability of the hydrogel in a highly swollen state. On the other hand, the presence of PVA increases the swelling capacity of the system.

### 3.2. FTIR Analysis

**Figure 4:**
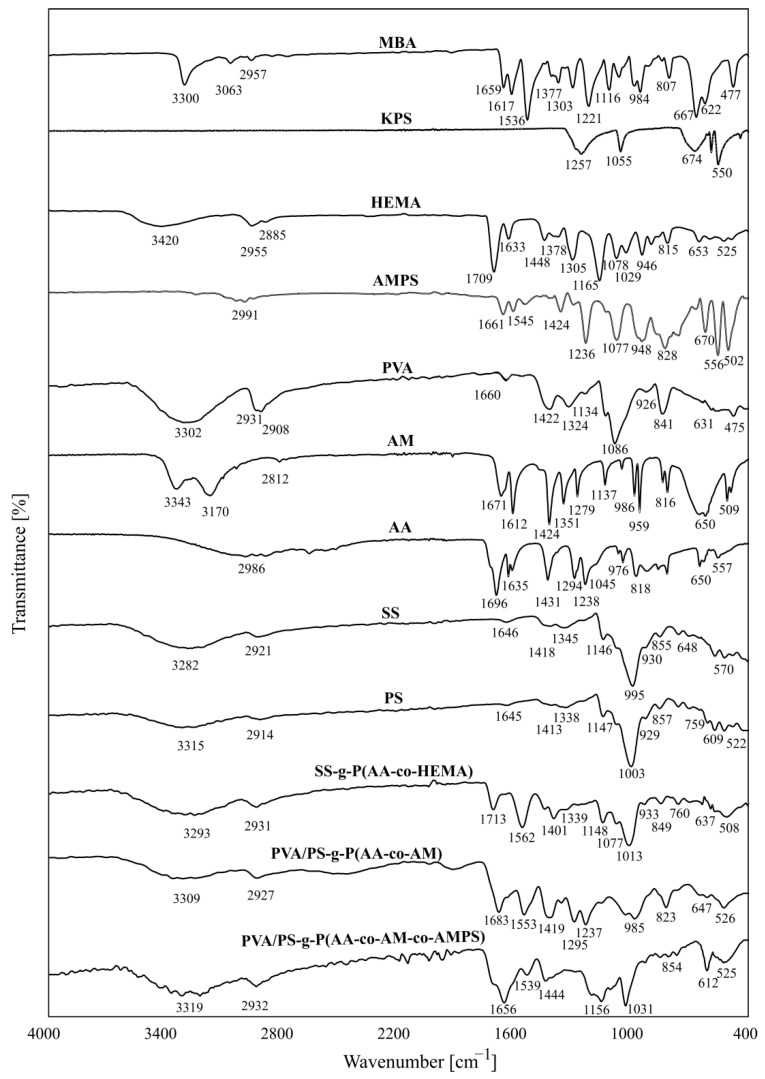
FTIR spectra of AMPS, PVA, AA, MBA KPS, PS, AM, PVA/PS-g-P(AA-co-AM-co-AMPS), PVA/PS-g-P(AA-co-AM) and SS-g-P(AA-co-HEMA).

The superabsorbent polymers obtained during this study are based on starch and acrylic acid, to which additional monomers have been added (AM, HEMA, AMPS). The authors analyzed the FTIR spectra of the products and main reagents (collected in Figure 4) based on the recent literature data and IR spectra interpretation handbooks [42]. From the obtained results one can notice that there are no noticeable differences between potato starch (PS) and soluble starch (SS), which was already discussed in the literature [12]. Both kinds of starch have spectra that show the same characteristic peaks and similar signal patterns. The peaks at 3300 and 2900 cm^−1^ are due to OH bonds and CH_2_ deformation, respectively [43]. The peaks at 1650 cm^−1^ can be assigned to flexing vibrations of water molecules absorbed in the amorphous regions of starch. The wavelength for about 1410 cm^−1^ is characteristic for the bending vibrations of C–H bonds in methyl groups, while the band about 1140 cm^−1^ indicates the presence of glycosidic C–O–C bonds. The peaks at about 1070 cm^−1^ and 1000 cm^−1^ can be assigned to starch crystalline and amorphous regions, respectively. The binding at about 930 cm^−1^ was attributed to the skeletal oscillation of the α 1–4 backbone linkages. The peak found at 760 cm^−1^ was assigned to the stretching of C–C bonds and the one at about 570 cm^−1^ was assigned to the skeletal vibration of the pyranose ring. All spectra with designated characteristic peaks are present in the Supplementary Materials.

The spectrum of the superabsorbent network SS-g-P(AA-co-HEMA) shows a characteristic peak of carboxyl group at 1713 cm^−1^ for the carboxylic group from acrylic acid and the ester group derived from 2-hydroxyethyl methacrylate [44]. The band in question results from the disappearance of the sharp peak at 1635–1633 cm^−1^ and 818–815 cm^−1^ (Supplementary fields, Figures S2, S10 and S12), which correspond to the bending vibration of the C=C and C–H bonds in the vinyl group of the monomer [20,45]. The bands found at wavelengths 1417 and 760 cm^−1^, correspond with the bending and rocking modes of CH_2,_ respectively. These bands are characteristic of acrylic polymers and confirm the successful formation of polyacrylane carbon backbone. The MBA cross-linker in the FTIR spectrum shows the signals characteristic for N–H groups at the wavelength of 3365 cm^−1^, the peak at the wavelength of 1654 cm^−1^ shows the C=O group, and the peak at 1338 cm^−1^ confirms the presence of the C=C groups.

The superabsorbent polymer abbreviated as PVA/PS-g-P(AA-co-AM) consists of polycarbonate backbone and grafts containing combination of two mers—AA and AM–crosslinked by MBA and from additional free PVA chains penetrating the acryl-starch network. The 0raw spectra of this system are included in the Supplementary Materials (Figures S2–S4, S6, S7 and S9). Figure 4 presents the spectra with the important bands idicated, where a broad band at 3309 cm^−1^ and it is attributed to the presence of a hydroxyl group that is hydrogen bonded to various degrees. The band at about 2927 cm^−1^ can be attributed to the variety of –CH_2_ stretching vibrations. The bands at the wavelengths 1683 and 1419 cm^−1^ indicate carboxyl groups. The peaks appearing at 1237 cm^−1^ in the spectrum can be attributed to the presence of the C–O–C moieties. The band at 1031 cm^−1^ can be assigned to a C–O stretching vibration [46]. The spectrum of the superabsorbent polymer shows the absorption peaks characteristic both of starch and of graft copolymerized acrylic acid and acrylamide. The peak at 1134 cm^−1^ for poly(vinyl alcohol) attributed to crystalline parts of the polymeric chains was shifted to a lower wavenumber [47]. This shift indicated the formation of hydrogen bonds between poly(vinyl alcohol), and hydrogen bond acceptors in moieties derived from acrylic acid and acrylamide. The C–O–C bond vibration absorption peak at 1147 cm^−1^ wavelength in starch was significantly reduced due to cross-linking [30]. The spectra presented confirmed the cross-linking of the PVA/SS-g-P(AA-co-AM) polymer.

In the spectrum of PVA/PS-g-P(AA-co-AM-co-AMPS) system peaks corresponding with characteristic groups present in the monomers are observed on polymer spectra with varied intensity, which confirm the incorporation of these monomers in the polymer network. The NH_2_ stretching band can be found at 3541–3221 cm^−1^. The band at 1652 cm^−1^ can be attributed to C=O stretching mode, bands at 1539 cm^−1^, and 1444 cm^−1^ are characteristic for NH_2_ groups. Antisymmetric vibrations of S–O bonds in SO_2_ group can be found at 1294 cm^−1^ and are accompanied by a band at 1031 cm^−1^ corresponding to symmetrical vibration for SO_2_ group [40]. The characteristic absorption bands of the sulfonate group at 1156, 1031 and 612 cm^−1^ can be observed in more details on spectra included in the Supplementary Materials (Figures S1–S4). In the spectra in Figure 4 also show the characteristic bands for aliphatic methylene moieties: 2932, 1539, 1414 cm^−1^ corresponding with the asymmetric stretching, asymmetric deformation and symmetrical deformation modes [48]. The presence of these characteristic bands confirms the graft copolymerization of AA, AM and AMPS to starch backbone.

### 3.3. Scanning Electron Microscope

**Figure 5:**
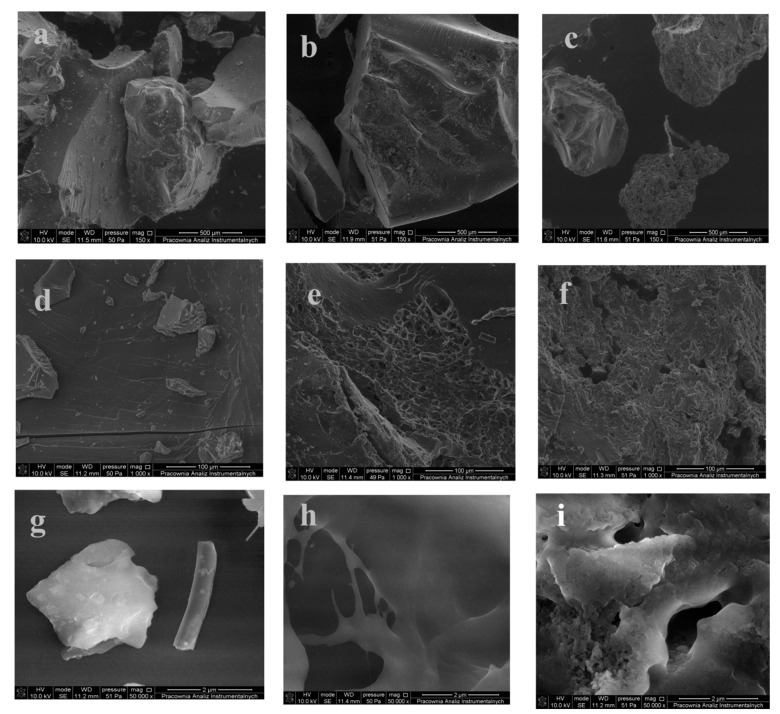
SEM images at 150× magnification of substances: (**a**) PVA/PS-g-P(AA-co-AM-co-AMPS), (**b**) PVA/PS-g-P(AA-co-AM), (**c**) SS-g-P(AA-co-HEMA), 1000× magnification of substances (**d**) PVA/PS-g-P(AA-co-AM-co-AMPS), (**e**) PVA/PS-g-P(AA-co-AM), (**f**) SS-g-P(AA-co-HEMA), 50,000× magnification of substances (**g**) PVA/PS-g-P(AA-co-AM-co-AMPS), (**h**) PVA/PS-g-P(AA-co-AM), (**i**) SS-g-P(AA-co-HEMA).

Scanning electron microscopy (SEM) was used to study the morphology of the synthesized samples. Photos a, d, g in Figure 5 show PVA/PS-g-P(AA-co-AM-co-AMPS) at a magnification of 150×, 1000×, 15,000×, respectively. The recorded samples show dense irregular granules with few cracks typical for polymers comminuted in a grinder. The photos show altered surface morphology compared to the samples of raw starch (smooth, oval pots) [12]. This proves that grafting polymerization significantly alters starch morphology. It is worth noting that most of the superabsorbent polymers reported in the literature have pores and spherical cracks, while our samples show linear cracks in seemingly compact grains. This suggests that the obtained systems are micro- and mesoporous. This kind of superabsorbent polymer is more mechanically stable than those having an extensive microporous structure. Microporous SAPs can be used in those industries where high mechanical strength is required. A good application could be agriculture, where polymers are placed deep into the soil and should withstand the associated pressure [49].

In the case of the PVA/PS-g-P(AA-co-AM) sample (photos b, e, h in Figure 5, enlarged by 150×, 1000×, 15,000× respectively), irregular, tightly bound granules have a porous surface with interconnected pores, which can guarantee excellent liquid absorption properties. The pores are culite-shaped with long channels extending into the sample. Presumably, the lattice is more flexible, which allows the liquid to expand more into a larger pore volume. The elongated pores indicate the direction of gas escape during foaming.

The three-dimensional network in the case of the polymer SS-g-P(AA-co-HEMA), shown in Figure 5c,f,i at a magnification of 150×, 1000×, 15,000×, respectively, is characterized by the most porous structure. The surface of the superabsorbent polymer is rough with numerous folds and micrograined aggregates. This polymer system, in contrast to that previously discussed, is not a SIPN kind of superabsorbent. The superabsorbent polymer obtained by the modification of starch with AA and HEMA monomers has a very rough structure with microscale interstitial spaces and numerous warts. The surface of the polymer shaped in this way helps to increase the size of the channels, folds and spherical pores, which will facilitate the diffusion and absorption of liquids and increase the speed of moisture penetration into the network, which will consequently ensure excellent swelling properties. All the images shown in Figure 5 are available in their original size in Supplementary Material Figures S38–S46.

By adjusting several factors, pores of a certain size can be produced, including porosity, type and amount of surfactant, amount of solvent, type and amount of inert gas. In the case of superabsorbent polymers, the size and number of pores play a key role in the water absorption and sorption capacity and the rate of liquid absorption by reducing the transport resistance [50]. Three methods are used to create a porous structure: water-soluble porogens [51], foaming [52] and phase separation [53]. In this article, the pores formed are the result of heating in a vacuum.

### 3.4. Thermogravimetric Analysis

**Figure 6:**
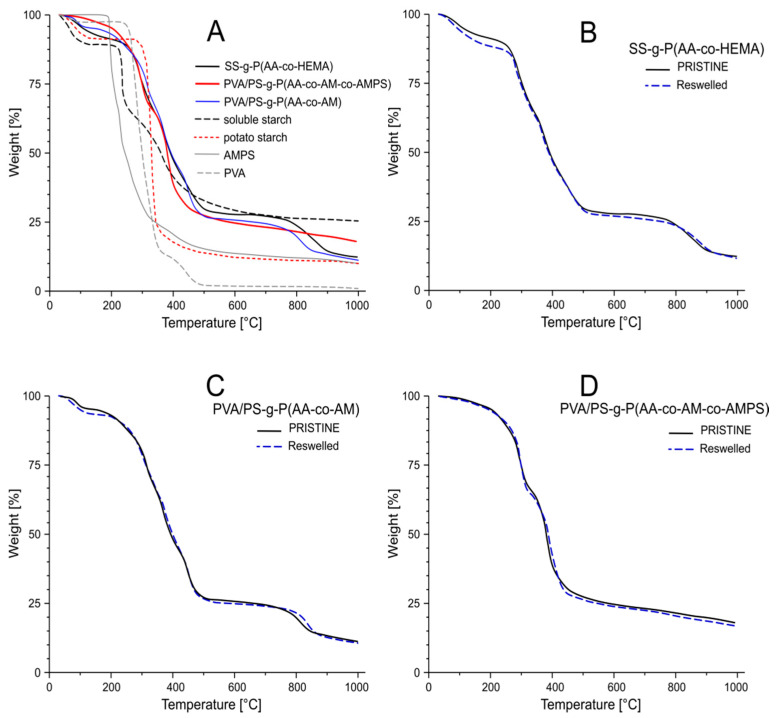
TG curves of studied substances superabsorbent polymers SS-g-P(AA-co-HEMA), PVA/PS-g-P(AA-co-AM-co-AMPS), PVA/PS-g-P(AA-co-AM) and selected starting compounds soluble starch, potato starch, AMPS and PVA (**A**). Comparison of polymer TG curves before swelling and after 10 swelling/drying cycles for SS-g-P(AA-co-HEMA) (**B**), PVA/PS-g-P(AA-co-AM) (**C**) and PVA/PS-g-P(AA-co-AM-co-AMPS) (**D**).

Thermal stability is one of the basic features of hygienic materials. This is important not only during use or storage, but also when materials come into contact with the human body. This makes sense especially when a new component with different thermal properties is added to enhance an already used material. Thermogravimetric analysis is the most commonly used technique to study thermal stability. In our work, it was applied to analyze the influence of the chemical composition of the grafts attached to starch on the thermal stability of the starch-based superabsorbent polymers. Thermogravimetric analysis of the superabsorbent polymers and their starting components was performed to assess their degradation profile and thermal stability (Table 1 and Table 2 and Figure 6). In Figure 6B–D are shown juxtapositions of the TG plots obtained for pristine material and material after 10 swelling/drying cycles. The presented plots indicate that periodic swelling and drying does not influence thermal stability of the synthesized materials. This aspect is important in the context of their future application as component of hygienic materials, which may be subjected to high temperature treatment (e.g., during sterilization). The components of the individual superabsorbent polymers were also tested for comparative purposes on the basis of the initial decomposition temperature, the percentage of weight loss at various stages of decomposition, and the percentage of residual mass at the maximum decomposition temperature.

Soluble starch (SS) showed a characteristic three-stage thermogram (Supplementary material, Figure S35), where the main weight loss (78%) occurred in the second stage. On the other hand, the potato starch (PS) shows the maximum decomposition temperature at the second stage, about 313.9 °C. However, the third stage of degradation for the SS was relatively slow; the degradation was almost complete at 361.8 °C.

Thermogravimetric analysis of SS-g-P(AA-co-HEMA) (Figure 6A, Table 1 and Table 2) reveals that the weight loss occurs in five steps. The first stage in the temperature ranges from 45 °C to 190 °C and corresponds with 3% weight loss, which can be explained by a loss of adsorbed remains of residual water and water bound physically (hydrogen bonds). The next stage shows loss of 21.88% of the weight and span from about 200 °C to 330 °C. This stage can be attributed to the breaking of C–O–C bonds in the starch main chain and dehydration of saccharide rings [54,55]. The third and fourth steps ranging from 335 °C to 520 °C characterize the terminal degradation of the various structure of the graft branches composed of the carboxyl groups and the acrylic chains of poly(acrylic acid) and 2-hydroxyethylmethacrylate [45,55]. The appearance of these steps indicates successful modification of the structure of the soluble starch chains, due to the grafting of the AA and HEMA chains [56]. The resulting polymer showed less weight loss than pure starch (Table 2). This means that starch grafting increases the thermal stability of the starch cross-linked products to some extent.

The degradation of PVA/PS-g-P(AA-co-AM-co-AMPS) has five steps (Figure 6B and Supplementary material, Figure S35). The first decomposition step represents a water evaporation process with a weight loss of 3 wt% ranges from 35 °C to 180 °C. The next phase shows a weight loss of 9 wt% and occurs in temperature range from 190 to 245 °C. This stage is characterized by dehydration of the saccharide rings and breaking the C–O–C bonds in the starch chain [54]. The third stage with a weight loss of 25 wt% was found in the temperature range from 250 °C to 330 °C and may be caused by the oxidation of PVA vinyl backbone to CO_2_ and might be acceptable evidence for the decomposition of PVA [57]. The next step with the greatest weight loss of 51 wt% at 379.4 °C can be attributed to thermal degradation of acrylic graft branches and the removal of the water molecule from adjacent carboxyl groups. This process results in the breaking of the grafted chains and the formation of an anhydride accompanying the destruction of the cross-linked polymer structure [58]. The fifth step occurring at 810.7 °C with a weight loss 78 wt% may be the result of the removal of the SO_2_ molecule from AMPS counterparts of the outer chain attached to the polymer network [6].

The thermal properties of the PVA/PS-g-P(AA-co-AM) superabsorbent were shown in Figure 6C and assessed by means thermogravimetric analysis (TGA/DTA). The hydrogel thermogram shows the greatest number of decomposition steps, as many as ten. A weight loss of 5 wt% is attributed to the evaporation of absorbed and bound water (Table 1). The degradation of the polymer in the first five steps showed an approximate weight loss of 16 wt% over the temperature range from about 30 °C to 280 °C, which can be attributed to the dehydration and breakdown of the starch particles [59]. The next steps correspond to the degradation of the starch polymer chain, observed in the range of 290 °C–500 °C with a total weight loss of about 66 wt%. A decay above 500 °C giving 80 wt% weight loss can be attributed to the degradation of the polymer chains and acrylate cross-linked strains, showing that the thermal stability of the starch copolymers is higher than that of the native starch [12]. The addition of synthetic monomers to the starch improves its thermal stability. This may be attributed to the generation of the new chemical bonds [55].

On the basis of the discussed TGA thermograms, it was found that the temperature at which 5 and 10 wt% weight loss (Table 1) occurred increased for the obtained superabsorbent polymers as compared to the data for individual monomers. These results show that the thermal stability of the superabsorbent composites, at this point, was lower compared to the single monomers. In the case of 50 wt%, the decomposition temperatures for the superabsorbent polymers are much higher than for the reference materials, which proves a better thermal stability of the samples obtained. This improvement in the thermal stability of the polymers can be attributed to the marked interaction between the monomers concerned and the polymer matrix.

The thermal stability of the superabsorbent polymers can be determined from the initial decomposition temperature (T_onset_) given in Table 2. All the polymers obtained show a lower T_onset_ compared to the starting monomers. The results of the TG show that there was an increase in the thermal stability of the materials obtained, which confirms the cross-linking of the chains. All the results for the superabsorbent polymers in Table 2 confirm that the obtained materials show better thermal stability than the reference materials, which may increase the use of the resulting products.

### 3.5. Swelling Properties of Superabsorbent Polymers

#### 3.5.1. Analysis of Water Absorbency

**Figure 7:**
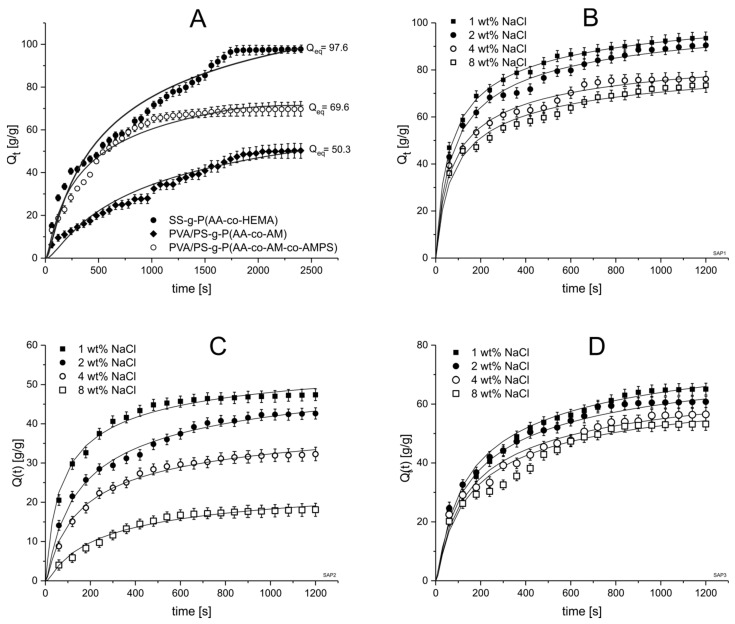
Water absorbency plot of studied polymers in water (**A**) and in different NaCl solutions plotted separately for each polymer: SS-g-P(AA-co-HEMA) (**B**), PVA/PS-g-P(AA-co-AM) (**C**) and PVA/PS-g-P(AA-co-AM-co-AMPS) (**D**).

Superabsorbent polymers used in hygiene products, and especially in disposable diapers, must be characterized by high water retention capacity and high water retention [60,61]. These properties increase when the material has the ability to draw water into the polymer matrix, which means a porous structure with many fractures, capillaries and space for the absorbed fluid. To investigate the effect of structural variability on the water absorption efficiency of the produced hydrogels, the amount of water absorbed at equilibrium was studied. The equilibrium swelling Q_eq_ (g/g) of hydrogel was measured based on the temporal evolution of swelling degree Q_t_ (g/g) deionized water (pH = 6.9 ± 0.1) at 25 °C. Based on the plot Q_t_ = *f*(t) (Figure 7) the mathematical model described by Equation (4) was fitted by means of the least squares technique and the value of retardation time (τ) was calculated using the transformed form of Equation (3). A similar procedure was applied to interpret the swelling data in other media. The results of the analysis are shown in Table 3.

The water absorption characteristics of all superabsorbent polymers with different monomer content are shown in Figure 7A. Based on the collected data, it can be observed that the water absorption increases with the immersion time of all samples. The water absorption curves increase quickly during the first few minutes then equilibrate over time and the line flattens out. This type of plot is characteristic for swelling dynamics and indicates a fast uptake of water at the start of the dive, then slowing down near the saturation.

Based on the graph, we can observe that the highest uptake rate and Q_eq_ are in case of SS-g-P(AA-co-HEMA). This polymer consists of chemically crosslinked graft modified starch without physically interpenetrating additive. The other two polymers have a structure of interpenetrating network of modified starch and PVA. The latter occupies free spaces in a crosslinked starch-acrylane network preventing the system from reaching a high degree of crosslinking. Theoretically, when the crosslinking degree is lower, the water absorbance is higher. In our case, the SAPs composed of interpenetrating networks show significantly lower Q_eq_ as well as swelling rate at initial stage. This could be explained assuming that the physical interactions between grafted starch chains and PVA are so strong that they prevent easy water uptake.

Comparing the obtained results of the equilibrium swelling for potato starch (Q_eq_ = 0.25 [g/g]) and soluble starch (Q_eq_ = 0.90 [g/g]) with the results obtained for the resulting superabsorbent polymers, it can be concluded that, as expected, the starch samples do not swell at room temperature and do not retain deionized water. The native starch is insoluble in cold water and most organic solvents, which is especially related to the presence of an insoluble or sparingly soluble amylose fraction (one of which is water). Swelling is an exothermic process during which water molecules are absorbed into the amorphous zone where they are hydrogen bonded to free hydroxyl groups of glucose units in the polymer chains. However, if the aqueous starch suspension is heated above a certain temperature, the starch grains swell spherically and become amorphous. The above thermal transformation is called starch gelatinization and the temperature of this transition is called the gelatinization temperature.

In our previous research it has been confirmed that starch-based superabsorbent polymers can be modified using acrylic acid and this strengthens polymer networks [12]. One can assume that the deionized water absorption mechanism in recent materials is similar to the one described previously. Starch’s glucose units contain three hydroxyl groups capable of interacting with water molecules, but are also susceptible to hydrogen bond formation. The second property is responsible for the denser dry state of the polymer but it can also slow down the water absorption process. The graft chains play two important roles in these polymers. Firstly, they substantially increase the interchain distances between amylose and amylopectin chains. The importance of this effect is vital because densely packed polysaccharide chains form strong interchain interactions preventing the system from swelling. Increasing interchain distances facilitates the migration of the solvent through the material. The second aspect concerns the presence of a large number of hydrophilic functional groups, i.e., –COOH, –NH_2_, –SO_3_H, –CO(NH_2_)–. These groups have to be solvated. The ion solvation process requires a large amount of water inside polymer network. Moreover, since some of these ions are fixed to the polymer network, they cannot leave the polymer through leaking. These ions need to be accompanied by relevant counter ions with their solvation spheres. Summing up, ions produced through this dissociation result in an increase in water absorption [62]. Increasing the cross-link density can limit the molecular movement of the SAP chains, which limits the penetration of liquids into the polymer system and thus reduces the deionized water absorption capacity [63].

Figure 7A shows the swelling kinetics of all analyzed superabsorbent polymers in deionized water. The amount of water absorbed by each polymer increased gradually over time until the maximum value was obtained. The swelling increased over time, but after some time it reached its maximum value. This stage is known as equilibrium swelling. Upon contact of the sample with deionized water, the sample swells due to the solvation of ionic groups and hydroxyl groups at the polymer chain. When the hydrogel sample comes into contact with water, the water diffuses into pre-existing or dynamically formed spaces between macromolecular chains and interacts with specific parts of polymer network increasing interchain distances. The water diffusion mechanism in the hydrogel system is significant because it controls the rate of water transfer from environment to superabsorbent where it can be adsorbed. It is important in the hygienic industry, agriculture, biomedicine and environmental protection.

The results obtained for the rate parameter for the analyzed superabsorbent polymers show that deionized water absorption rate decrease in series PVA/PS-g-P(AA-co-AM-co-AMPS) (333.33 s), PVA/PS-g-P(AA-co-AM) (544.28 s), and SS-g-P(AA-co-HEMA) (1000.00 s). The data indicate univocally that semi-interpenetrating network hydrogels formed by incorporation of PVA chains to the system reach the saturation level of swelling faster than that composed only of graft modified starch [64]. The swelling kinetics are complicated due to the number of variables that need to be considered. A few of the most important to mention include the complex three-dimensional structure of the polymer network, the specific interaction between the ionophore groups and both the polymer pendant groups and the swelling medium modules, and the conformational freedom of the polymer chains [65]. In deionized water, polymer–water interactions predominate over polymer–polymer interactions, which allows for the swelling. However, the formation of polymer water adducts usually requires the breaking down of the initial polymer-polymer adducts. This process has a significant influence on swelling kinetics.

#### 3.5.2. Effect of Various pH Solutions on Swelling Behaviors

**Figure 8:**
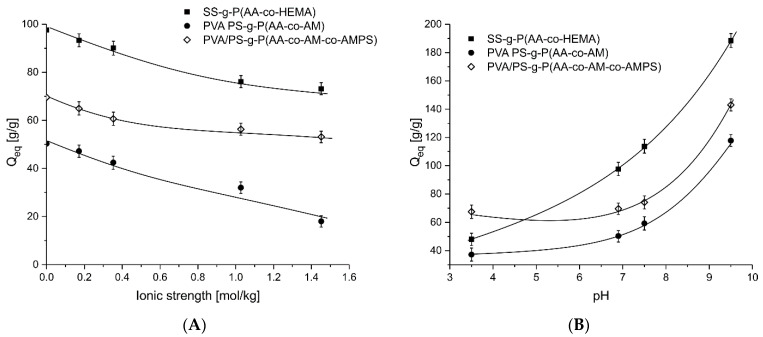
Dependence of hydrogels swelling on ionic strength (**A**) and pH (**B**).

An important issue in the swelling of superabsorbent polymers is their sensitivity to the ionic strength and pH. This comes from a large number of ionogenic groups present in the acrylic graft chains. The carboxylic group at low pH are associated and their degree of dissociation increases with pH. Dissociated ionophere groups attract more water to create solvation spheres, stabilizing the ion charge. This process strongly influences water uptake. Another important aspect is related to ions’ interchange between the gel polymer matrix and the surrounding medium, which is closely related to the ionic strength of the latter. In the study, the pH of the solvent was controlled with a pH meter by ChemLand, model 7011-01, with conductivity electrode and adjusted with 0.1 M NaOH and HCl solutions. All three samples of polymers swelling showed a clear sensitivity to a change in pH, which is demonstrated on Figure 8B.

Based on the obtained results, presented in Figure 8A,B, we can observe the increasing equilibrium swelling with higher pH. Moreover, it is evident that at lower pH (below 7) the influence is weak, while in the alkalic region Q_eq_ steeply raises. This can easily be attributed to influence of pH on the dissociation of ionic groups. When the pKa for a given ionic group is higher than the pH of the solution, the group is associated and does not contribute largely in the water uptake process [40]. In all the polymers the carboxylic group are present in large amounts and their attribution is significant at higher pH. However, there are also –SO_3_H groups present in AMPS counterparts of PVA/PS-g-P(AA-co-AM-co-AMPS) and they are responsible for higher water absorption compared to PVA/PS-g-P(AA-co-AM). Additionally, the acidic environment causes the stiffening of the polymer chains and as a result, of the protonation of sulfonic and carboxylic anions and the formation of hydrogen bonds between carboxylic carbonyl oxygen and the amide group of the acrylamide moieties, which causes the superabsorbent to contract. The anion-anion interaction (repulsion) increased the absorption properties (increased space between the chains) and the degree of ionization of the –COOH and –SO_3_H groups with the increase in the acidity of the solution [66]. The increase in anion density itself improves the absorption properties of the material, which results in an increase in the hydrophilicity of the superabsorbent polymers, facilitating the diffusion of the solution into the molecule [67].

#### 3.5.3. Effects of Saline Solutions on Swelling Behaviors

When developing materials for potential applications in hygienic products, the absorption of different concentrations of salt solutions is a very important parameter. In this section the superabsorbent polymers’ absorbency of NaCl_aq_ solutions of 1 wt%, 2 wt%, 4 wt% and 8 wt% were analyzed. The properties of the external solution, such as charge valence and salt solution concentration, have a great influence on the swelling of superabsorbent polymers [68]. Figure 8A,B show the results of the liquid absorption and it can be seen that the equilibrium absorption of the salt by the superabsorbent polymers in the NaCl_aq_ solution clearly decreases with increasing concentration. The shrinkage of the products is largely due to the fact that with an increase in the concentration of the external salt solution, the difference in osmotic pressures decreases, and the influence of the penetrating Na^+^ counterions on the anionic groups (COO^−^, SO_3_^−^) weakens the anion–anion repulsion between the carboxylate groups, thus reducing the swelling capacity [69]. The highest absorption values of the NaCl_aq_ solution were recorded for SS-g-P(AA-co-HEMA) reaching Q_eq_ of 93.3 (see Table 3) in 1 wt% NaCl_aq_ solution. For all three polymers the sorption of the NaCl_aq_ solution is lower than the sorption of pure water, and it decreases with the salt concentration. This is clearly seen from the graphs in Figure 7B–D, where the swelling dynamics is plotted for each polymer in every NaCl concentration. Additionally, in Table 3 the numerical parameters of the swelling curves and the experimental estimation of Q_eq_ are shown.

The research confirms the conclusion that all monovalent cationic forms with the same concentrations have a similar effect on the acrylate-based SAP capacity. The absorption capacity decreased several times (three to four times less) in the presence of salt ions compared to the absorption in deionized water. As already mentioned, the decrease in absorbency could result from a decrease in the osmotic pressure difference (ionic pressure) between the polymeric gel and the external solution, as the concentration of mobile ions between the gel and the aqueous phase decreases [70].

In order to investigate the effect of NaCl concentration on the swelling kinetics of the superabsorbent polymers in question, the increase in adsorbed mass over time was measured, and the swelling kinetics of the samples were plotted in Figure 7. As with deionized water, there was a sharp increase in sample swelling in the initial few minutes, and over time the line flattened approaching the equilibrium value. The superabsorbent polymers show sensitivity to NaCl solution concentration, due to the ionic strength influence on the dissociation equilibrium of both the deprotonable –COOH and protonable –NH_2_ groups. The influence of ionic strength on the equilibrium swelling is shown in Figure 8A. The presented points correspond to the experimental value, while the lines show only a general trend and do not reflect any reasonable theoretical model. The general trend is the same for all studied absorbents, i.e., the absorbency decreases with the increase in the solution’s ionic strength. The highest Q_eq_ values were found for polymer without additive of interpenetrating PVA (SS-g-P(AA-co-HEMA)). In the case of polymers with additional PVA, the system PVA/PS-g-P(AA-co-AM-co-AMPS) shows better swelling parameters due to presence of –SO_3_H groups.

## 4. Conclusions

Every day, research is carried out in many research centers to create a biodegradable superabsorbent that will meet the requirements of laws, regulations, and the demands of producers as well as consumers for disposable hygiene products. The biggest challenge is to obtain the products’ excellent absorption properties, which is not easy when they must also be biodegradable. The presented research is part of a large project whose main goal is to obtain a disposable hygiene product to reduce the amount of plastics ending up in landfill.

By grafting polymerization, two superabsorbent polymers were synthesized based on starch: SS-g-P(AA-co-HEMA), and starch + PVA interpenetrating polymer networks: PVA/PS-g-P(AA-co-AM-co-AMPS), PVA/PS-g-P(AA-co-AM). The polymers were grafted using a set of acrylic monomers such as acrylic acid (AA), 2-hydroxyethylmethacrylate (HEMA), poly(vinyl alcohol) (PVA), acrylamide (AM), and 2-acrylamido-2-methylpropane sulfonic acid (AMPS). The reactions were initiated with potassium persulfate and *N,N*′-methylenebisacrylamide was used as a crosslinker. Fourier Transform Infrared Spectroscopy (FTIR) and thermogarvimetric analysis (TA) confirmed the formation of the expected products and the cross-linking of chains. The examination of the morphology of the separated polymer grains using the scanning electron microscope (SEM) method confirmed that the obtained materials had a porous structure, which had a decisive influence on the absorption properties of individual polymers. SS-g-P(AA-co-HEMA) showed the greatest number of channels, spaces between layers and pores. Their positive impact on water uptake was confirmed by the results of swelling for deionized water. The pH has a significant influence on the swelling capacity; the more alkaline the environment, the higher the liquid absorption rate for all superabsorbent polymers tested. The highest value of Q_eq_ at pH 9.5 was found for the sample PVA/PS-g-P(AA-co-AM-co-AMPS) due to presence of sulfonic groups. The swelling capacity of the materials decreased with the increasing concentration of the Na^+^ ions. It can be assumed that the ionic repulsion between the charged groups incorporated into the gel matrix by external pH modulation is the main driving force responsible for the observed swelling characteristics. Analyzing the deionized water absorption rate parameter, the following series of absorbency was established: SS-g-P(AA-co-HEMA) > PVA/PS-g-P(AA-co-AM-co-AMPS) > PVA/PS-g-P(AA-co-AM), and the same absorbency decrease series was preserved in the salt solutions. Efforts to improve the swelling profile of superabsorbent polymers are still underway and we hope that the overall performance in terms of water absorption capacity and aqueous solutions will be improved by the further modification of similar hydrogel systems.

## Figures and Tables

**Table 1:** The mass loss results derived from thermogravimetric analysis (TGA).

Sample Code	TGA (5 wt% Loss) (°C)	TGA (10 wt% Loss) (°C)	TGA (50 wt% Loss) (°C)
SS	59.2	105.4	355.9
PS	70.6	280.2	315.8
KPS	295.5	472.7	—
MBA	198.5	210.7	250.2
AMPS	190.5	193.1	243.6
PVA	251.2	260.2	297.4
AM	107.4	119.6	282.1
PVA/PS-g-P(AA-co-AM-co-AMPS)	198.4	242.0	377.9
SS-g-P(AA-co-HEMA)	105.6	226.7	387.2
PVA/PS-g-P(AA-co-AM)	141.3	223.6	386.0

**Table 2:** TG data for monomers and superabsorbent polymers.

Sample Code	Temperature of the First Thermal Event after Water Evaporation (°C)	Sharp Decomposition Temperature in the Second Decomposition Stage (°C)	Maximum Decomposition Temperature (°C)	Maximum Weight Loss (%)
SS	229.5	361.8	229.5	78
PS	313.9	-	313.9	55
KPS	237.5	294.3	294.3	95
MBA	187.8	239.3	239.3	62
AMPS	194.0	225.9	193.4	89
PVA	268.6	431.1	268.9	80
AM	152.2	243.7	152.2	68
PVA/PS-g-P(AA-co-AM-co-AMPS)	135.9	235.6	379.4	49
SS-g-P(AA-co-HEMA)	289.5	372.2	372.2	55
PVA/PS-g-P(AA-co-AM)	213.2	241.9	356.9	60

**Table 3:** The collection of the results of fitting the experimental data to Equation (4) and values of parameter τ calculated from Equation (3).

Solution	A [g/g]	b [s^1/2^]	R^2^	FStdE	F	τ [s]	Q_eq_ [g/g]
**SS-g-P**(**AA-co-HEMA**)							
Water	158.86	23.58	0.965	4.86	499.5	1000.0	97.60
NaCl 1 wt%	115.15	7.16	0.999	0.84	13,213.1	208.3	93.30
NaCl 2 wt%	112.66	7.95	0.992	1.92	2400.7	222.2	90.11
NaCl 4 wt%	96.19	7.69	0.988	2.00	1614.1	227.3	76.07
NaCl 8 wt%	91.39	8.20	0.984	2.21	1181.3	243.9	73.11
**PVA/PS-g-P**(**AA-co-AM**)							
Water	99.78	33.78	0.970	2.57	842.1	588.2	50.30
NaCl 1 wt%	61.36	7.78	0.990	1.20	1866.1	153.9	47.25
NaCl 2 wt%	60.77	11.79	0.994	0.89	3071.1	196.1	42.48
NaCl 4 wt%	47.17	11.98	0.993	0.73	2770.8	208.3	32.04
NaCl 8 wt%	30.33	16.07	0.983	0.70	1128.5	227.3	17.99
**PVA/PS-g-P**(**AA-co-AM-co-AMPS**)							
Water	107.23	18.17	0.973	3.03	756.3	333.3	69.60
NaCl 1 wt%	92.00	11.50	0.996	0.93	3432.7	238.1	64.96
NaCl 2 wt%	86.15	11.52	0.999	1.52	1665.5	249.9	60.67
NaCl 4 wt%	79.13	11.54	0.984	1.76	1145.4	256.4	56.34
NaCl 8 wt%	76.27	11.73	0.999	1.10	1579.6	277.9	53.17

Q_eq_—is experimentally estimated equilibrium swelling.

## Data Availability

Not applicable.

## Supplementary Materials

The following are available online at: https://www.mdpi.com/article/10.3390/ijms22094325/s1, Figure S1: FTIR spectra of poly(vinyl alcohol)/potato starch-g-poly(acrylic acid-co-acrylamide-co-2-acrylamido-2-methylpropane sulfonic acid), Figure S2: FTIR spectra of acrylic acid (AA), Figure S3: FTIR spectra of acrylamide (AM), Figure S4: FTIR spectra of potato starch (PS), Figure S5: FTIR spectra of potassium persulfate (KPS), Figure S6: FTIR spectra of *N,N*′- methylenebisacrylamide (MBA), Figure S7: FTIR spectra of poly(vinyl alcohol) (PVA), Figure S8: FTIR spectra of 2-acrylamido-2-methylpropane sulfonic acid (AMPS), Figure S9: FTIR spectra of poly(vinyl alcohol)/potato starch-g-poly(acrylic acid-co-acrylamide), Figure S10: FTIR spectra of 2-hydroxyethylmethacrylate (HEMA), Figure S11: FTIR spectra of soluble starch (SS), Figure S12: FTIR spectra of soluble starch-g-poly(acrylic acid-co-2-hydroxyethylmethacrylate), Figure S13: TGA of *N,N*′-methylenebisacrylamide (MBA), Figure S14: DTA of *N,N*′-methylenebisacrylamide (MBA), Figure S15: TGA of potassium presulfate (KPS), Figure S16: DTA of potassium persulfate (KPS), Figure S17: TGA of potato starch (PS), Figure S18: DTA of potato starch (PS), Figure S19: TGA of soluble starch (SS), Figure S20: DTA of soluble starch (SS), Figure S21: TGA of soluble starch-g-poly(acrylic acid-co-2-hydroxyethylmethacrylate) before swelling, Figure S22: DTA of soluble starch-g-poly(acrylic acid-co-2-hydroxyethylmethacrylate) before swelling, Figure S23: TGA of poly(vinyl alcohol)/potato starch-g-poly(acrylic acid-co-acrylamide-co-2-acrylamido-2-methylpropane sulfonic acid) before swelling, Figure S24: DTA of poly(vinyl alcohol)/potato starch-g-poly(acrylic acid-co-acrylamide-co-2-acrylamido-2-methylpropane sulfonic acid) before swelling, Figure S25: TGA of poly(vinyl alcohol)/potato starch-g-poly(acrylic acid-co-acrylamide) before swelling, Figure S26: DTA of poly(vinyl alcohol)/potato starch-g-poly(acrylic acid-co-acrylamide) before swelling, Figure S27: TGA of poly(vinyl alcohol) (PVA), Figure S28: DTA of poly(vinyl alcohol) (PVA), Figure S29: TGA of 2-acrylamido-2-methylpropane sulfonic acid, Figure S30: DTA of 2-acrylamido-2-methylpropane sulfonic acid, Figure S31: TGA of poly(vinyl alcohol)/potato starch-g-poly(acrylic acid-co-acrylamide-co-2-acrylamido-2-methylpropane sulfonic acid) after swelling, Figure S32: DTA of poly(vinyl alcohol)/potato starch-g-poly(acrylic acid-co-acrylamide-co-2-acrylamido-2-methylpropane sulfonic acid) after swelling, Figure S33: TGA of poly(vinyl alcohol)/potato starch-g-poly(acrylic acid-co-acrylamide) after swelling, Figure S34: DTA of poly(vinyl alcohol)/potato starch-g-poly(acrylic acid-co-acrylamide) after swelling, Figure S35: The specific decomposition temperatures read from DTA plots of the polymers.
