# Supplementary material for: Synthesis and Characterization Superabsorbent Polymers Made of Starch, Acrylic Acid, Acrylamide, Poly(Vinyl Alcohol), 2-Hydroxyethyl Methacrylate, 2-Acrylamido-2-methylpropane Sulfonic Acid
Source: Int J Mol Sci. 2021 Apr 21;22(9):4325. doi: 10.3390/ijms22094325 (PMC8122658; doi:10.3390/ijms22094325)
Supplement: Supplementary file 1 [file ijms-22-04325-s001.zip › ijms-1156812-SI.docx]

Figure S1. FTIR spectra of poly(vinyl alcohol)/potato starch-g-poly(acrylic acid-co-acrylamide-co-2-acrylamido-2-methylpropane sulfonic acid).

Figure S2. FTIR spectra of acrylic acid (AA).

Figure S3. FTIR spectra of acrylamide (AM).

Figure S4. FTIR spectra of potato starch (PS).

Figure S5. FTIR spectra of potassium persulfate (KPS).

Figure S6. FTIR spectra of N, N’- methylenebisacrylamide (MBA).

Figure S7. FTIR spectra of poly(vinyl alcohol) (PVA).

Figure S8. FTIR spectra of 2-acrylamido-2-methylpropane sulfonic acid (AMPS).

Figure S9. FTIR spectra of poly(vinyl alcohol)/potato starch-g-poly(acrylic acid-co-acrylamide).

Figure S10. FTIR spectra of 2-hydroxyethylmethacrylate (HEMA).

Figure S11. FTIR spectra of soluble starch (SS).

Figure S12. FTIR spectra of soluble starch-g-poly(acrylic acid-co-2-hydroxyethylmethacrylate).


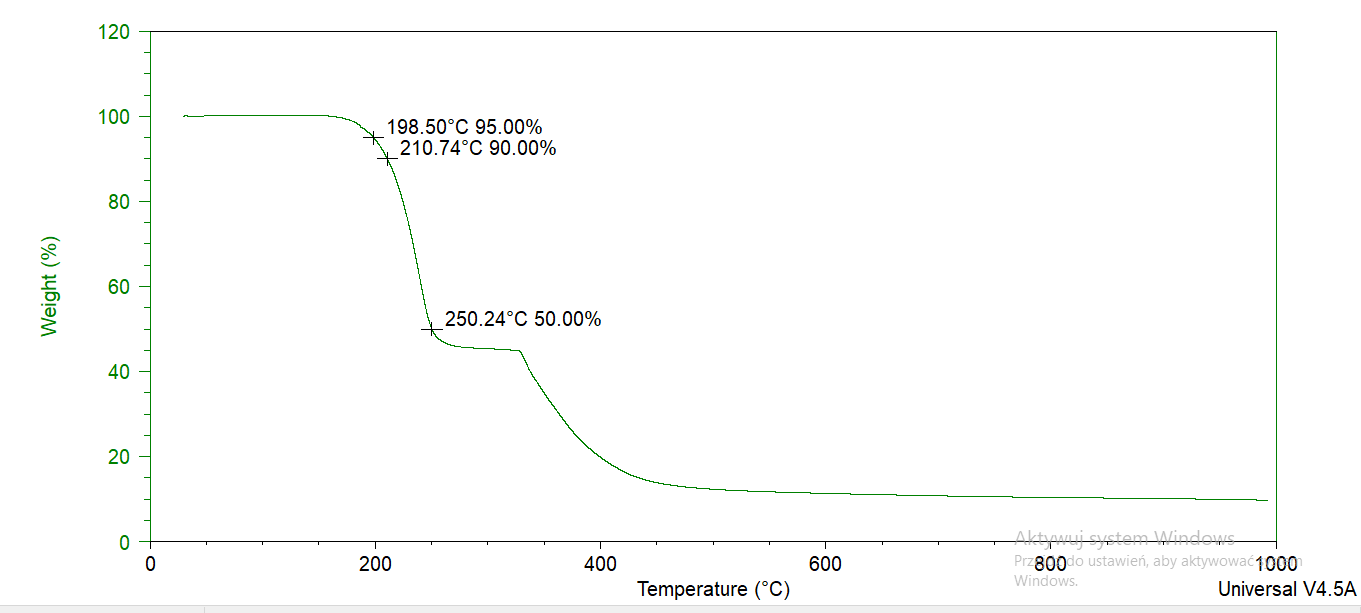


Figure S13. TGA of N, N’ - methylenebisacrylamide (MBA).


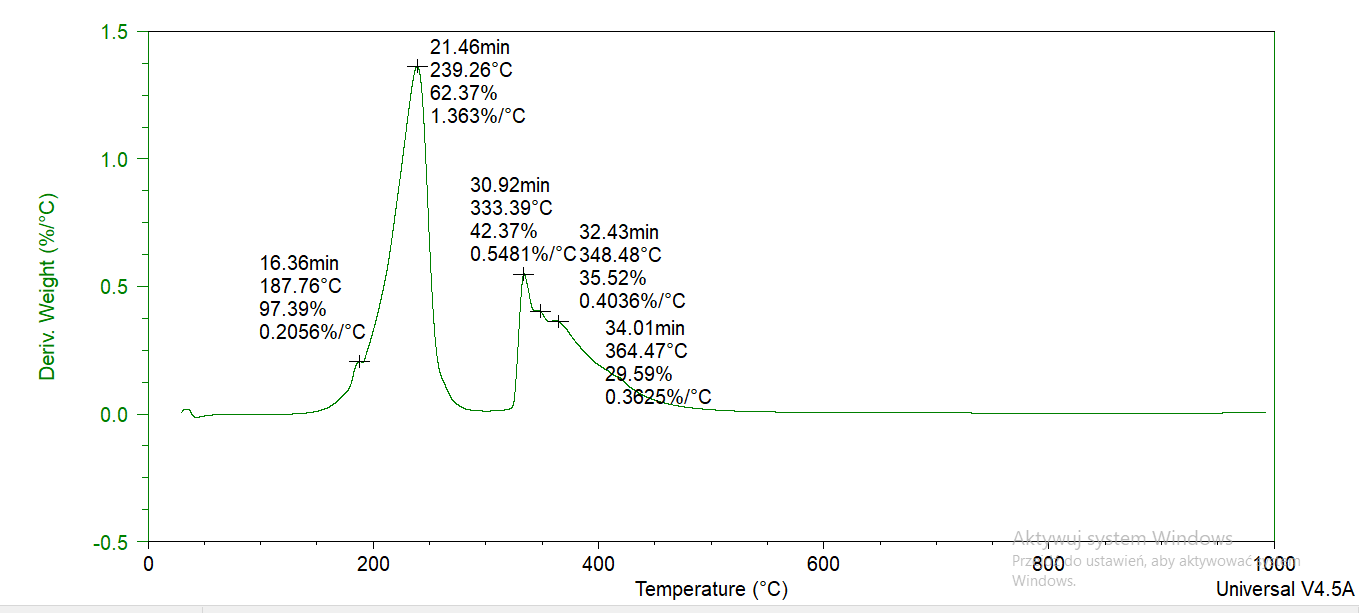


Figure S14. DTA of N, N’ - methylenebisacrylamide (MBA).


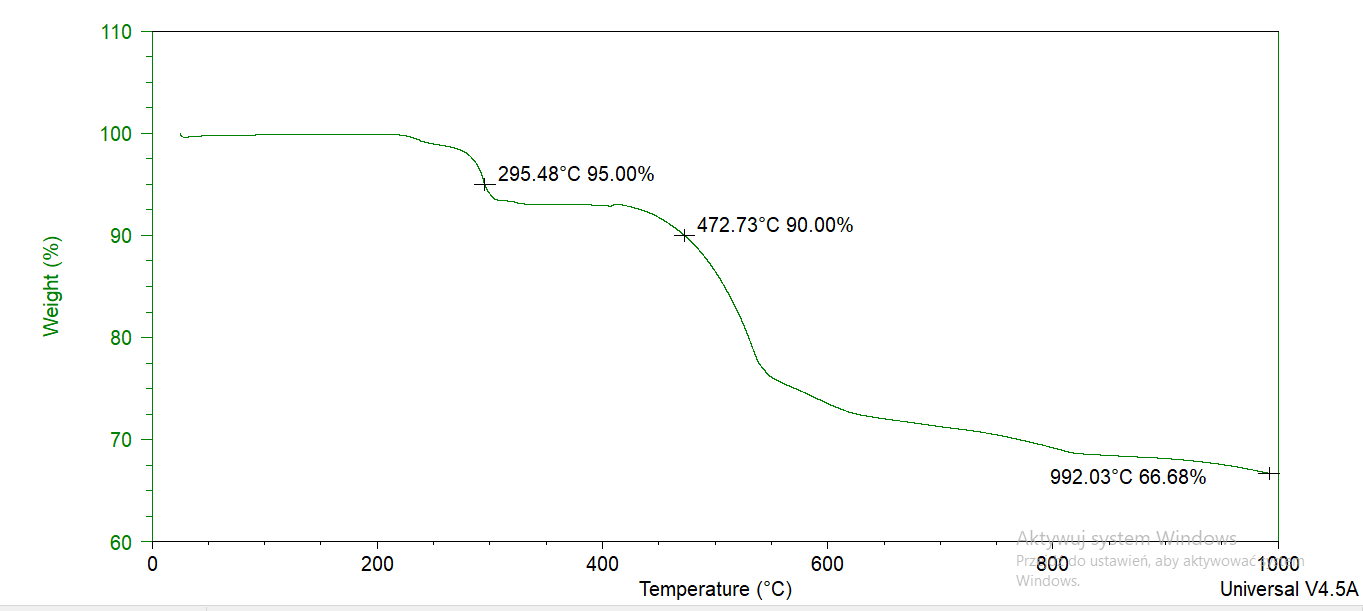


Figure S15. TGA of potassium presulfate (KPS).


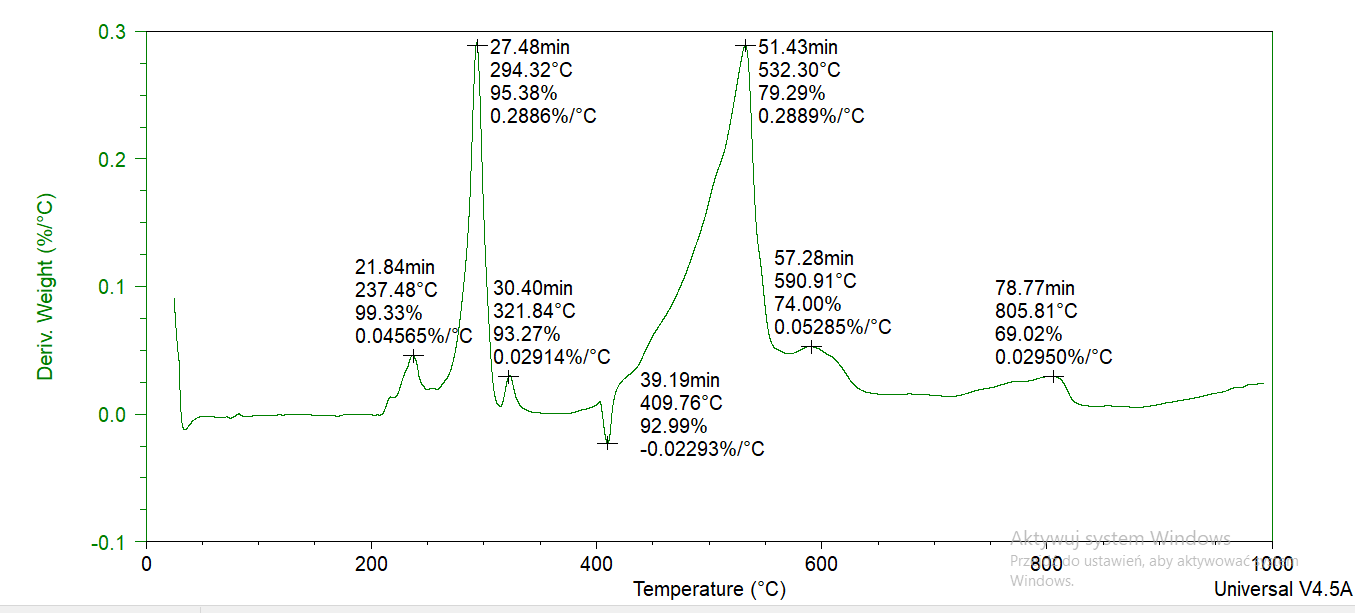


Figure S16. DTA of potassium presulfate (KPS).


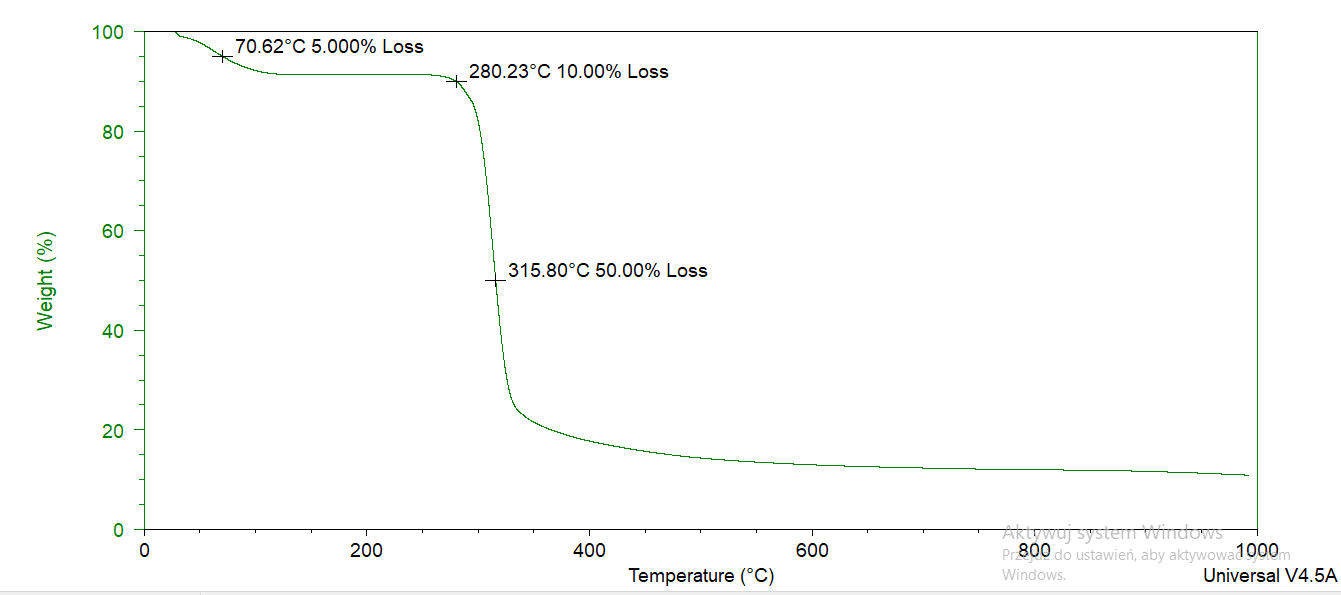


Figure S17. TGA of potato starch (PS).


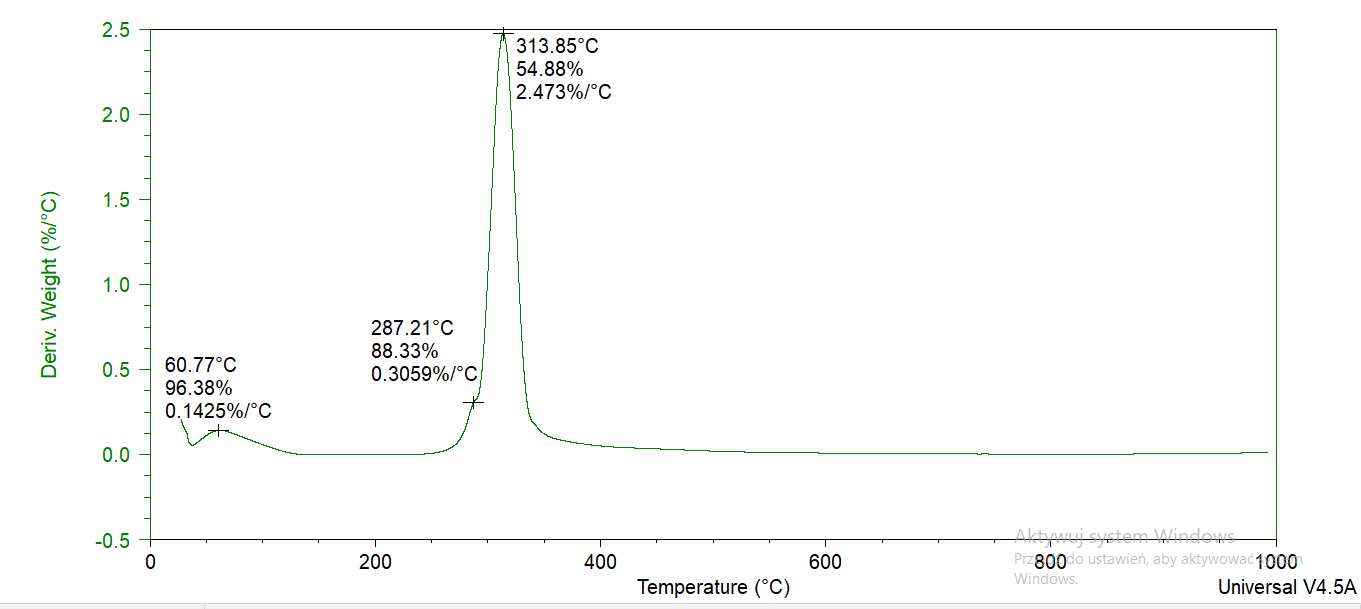


Figure S18. DTA of potato starch (PS).


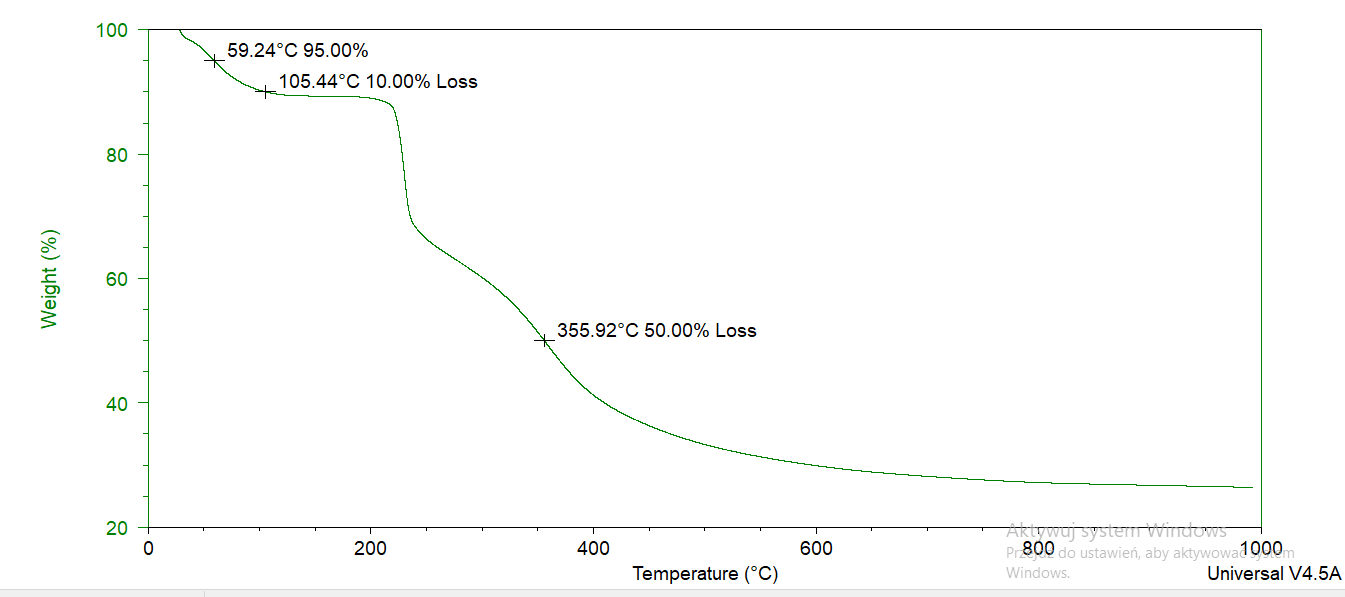


Figure S19. TGA of soluble starch (SS).


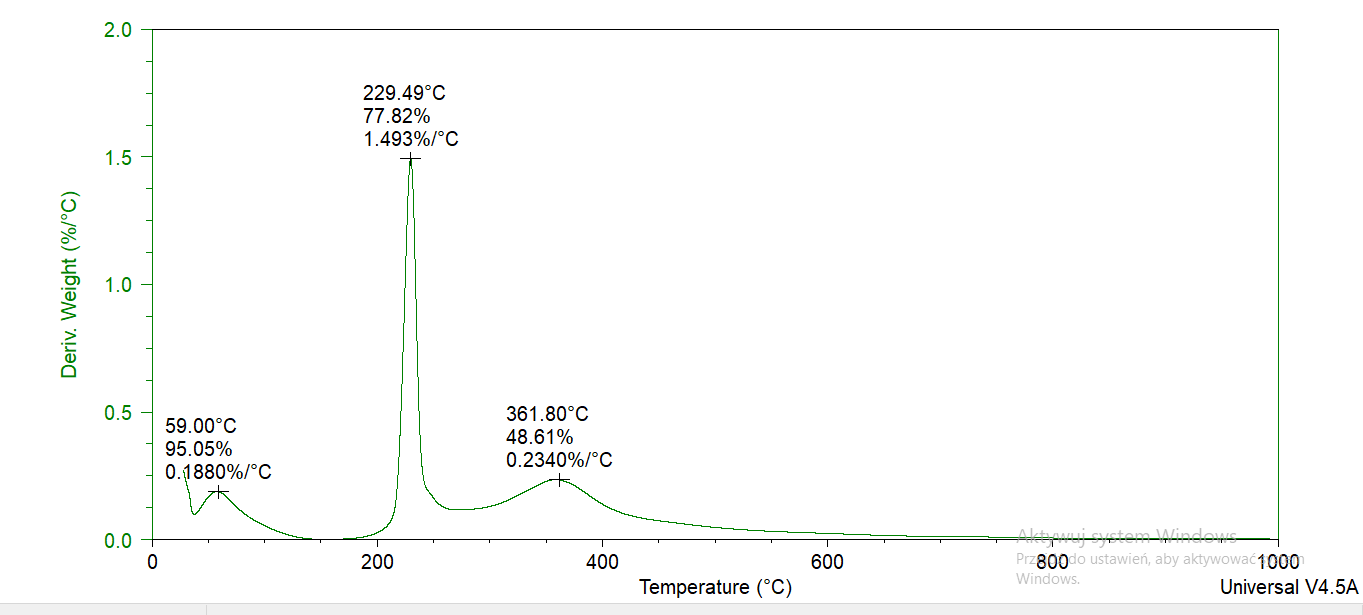


Figure S20. DTA of soluble starch (SS).

Figure S21. TGA of soluble starch-g-poly(acrylic acid-co-2-hydroxyethylmethacrylate) before swelling.

Figure S22. DTA of soluble starch-g-poly(acrylic acid-co-2-hydroxyethylmethacrylate) before swelling.

Figure S23. TGA of poly(vinyl alcohol)/potato starch-g-poly(acrylic acid-co-acrylamide-co-2-acrylamido-2-methylpropane sulfonic acid) before swelling.

Figure S24. DTA of poly(vinyl alcohol)/potato starch-g-poly(acrylic acid-co-acrylamide-co-2-acrylamido-2-methylpropane sulfonic acid) before swelling.

Figure S25. TGA of poly(vinyl alcohol)/potato starch-g-poly(acrylic acid-co-acrylamide) before swelling.

Figure S26. DTA of poly(vinyl alcohol)/potato starch-g-poly(acrylic acid-co-acrylamide) before swelling.

Figure S27. TGA of poly(vinyl alcohol) (PVA).

Figure S28. DTA of poly(vinyl alcohol) (PVA).

Figure S29. TGA of 2-acrylamido-2-methylpropane sulfonic acid.

Figure S30. DTA of 2-acrylamido-2-methylpropane sulfonic acid.

Figure S31. TGA of poly(vinyl alcohol)/potato starch-g-poly(acrylic acid-co-acrylamide-co-2-acrylamido-2-methylpropane sulfonic acid) after swelling.

Figure S32. DTA of poly(vinyl alcohol)/potato starch-g-poly(acrylic acid-co-acrylamide-co-2-acrylamido-2-methylpropane sulfonic acid) after swelling.

Figure S33. TGA of poly(vinyl alcohol)/potato starch-g-poly(acrylic acid-co-acrylamide) after swelling.

Figure S34. DTA of poly(vinyl alcohol)/potato starch-g-poly(acrylic acid-co-acrylamide) after swelling.

|  | Sample code | SS | PS | KPS | MBA | AMPS | PVA | AM | PVA/PS-g-P(AA-co-AM-co-AMPS) | SS-g-P(AA-co-HEMA) | PVA/PS-g-P(AA-co-AM) |
| --- | --- | --- | --- | --- | --- | --- | --- | --- | --- | --- | --- |
| Decomposition temperature [^o^C] | Step 1 | 59.0 | 60.8 | 237.5 | 187.8 | 194.0. | 62.6 | 89.2 | 135.9 | 79.3 | 77.5 |
|  | Step 2 | 229.5 | 313.9 | 294.3 | 239.3 | 226.0. | 268.6 | 152.2 | 235.6 | 289.5 | 213.2 |
|  | Step 3 | 361.8 | - | 321.8 | 333.4 | 244.6 | 431.1 | 243.7 | 296.7 | 372.2 | 241.9 |
|  | Step 4 | - | - | 532.3 | 348.5 | 257.9 | - | 295.2 | 379.4 | 450.4 | 259.0. |
|  | Step 5 | - | - | 590.9 | 364.5 | - | - | 396.4 | 810.7 | 852.0 | 272.5 |
|  | Step 6 | - | - | 805.8 | - | - | - |  | - | - | 356..9 |
|  | Step 7 | - | - | - | - | - | - | - | - | - | 408.2 |
|  | Step 8 | - | - | - | - | - | - | - | - | - | 435.1 |
|  | Step 9 | - | - | - | - | - | - | - | - | - | 451.3 |
|  | Step 10 | - | - | - | - | - | - | - | - | - | 796.3 |

Figure S35. The specific decomposition temperatures read from DTA plots of the polymers.
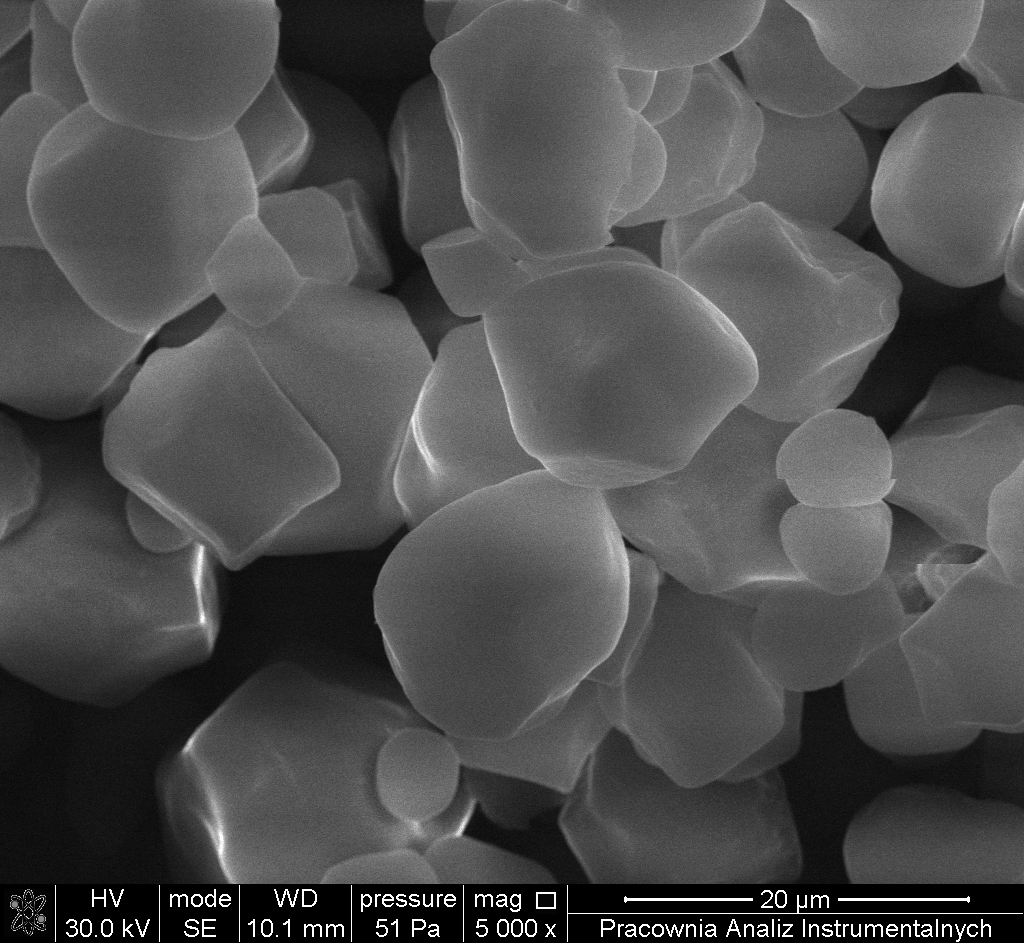


Figure S36. SEM images at 5 000× magnification of potato starch (PS).


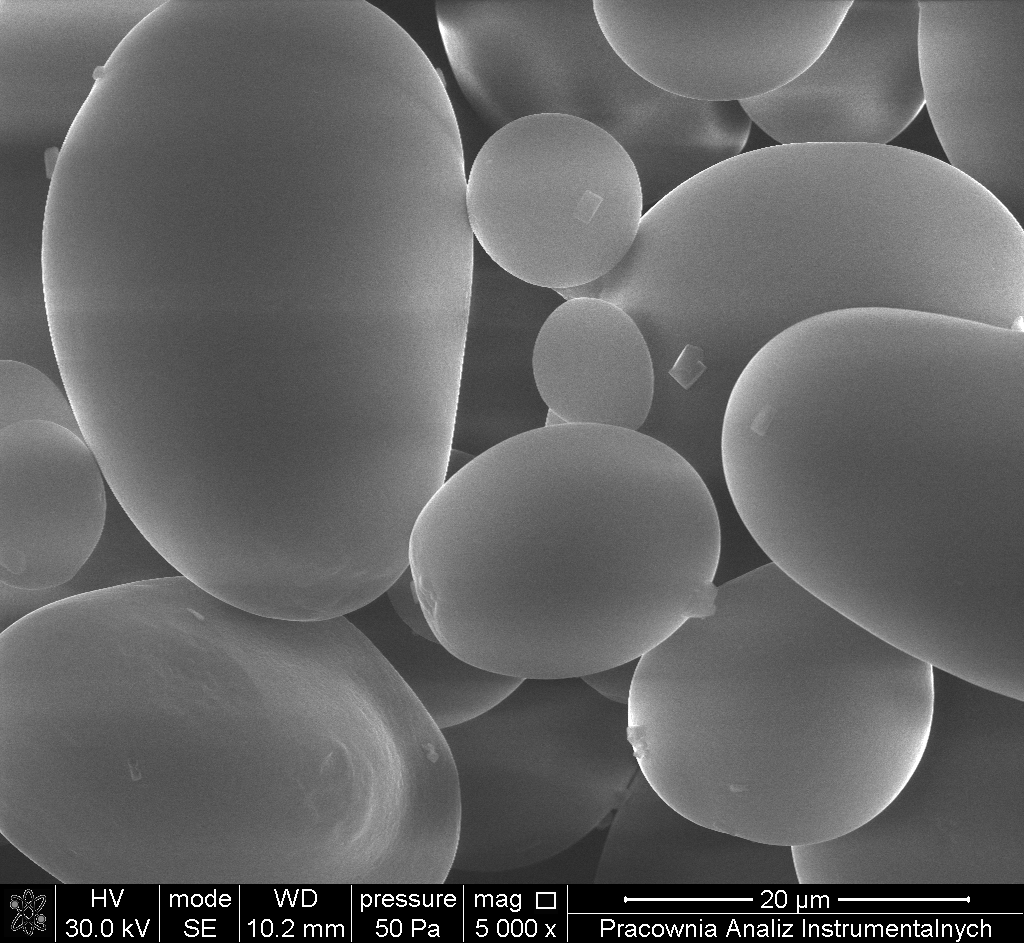


Figure S37. SEM images at 5 000× magnification of soluble starch (SS).


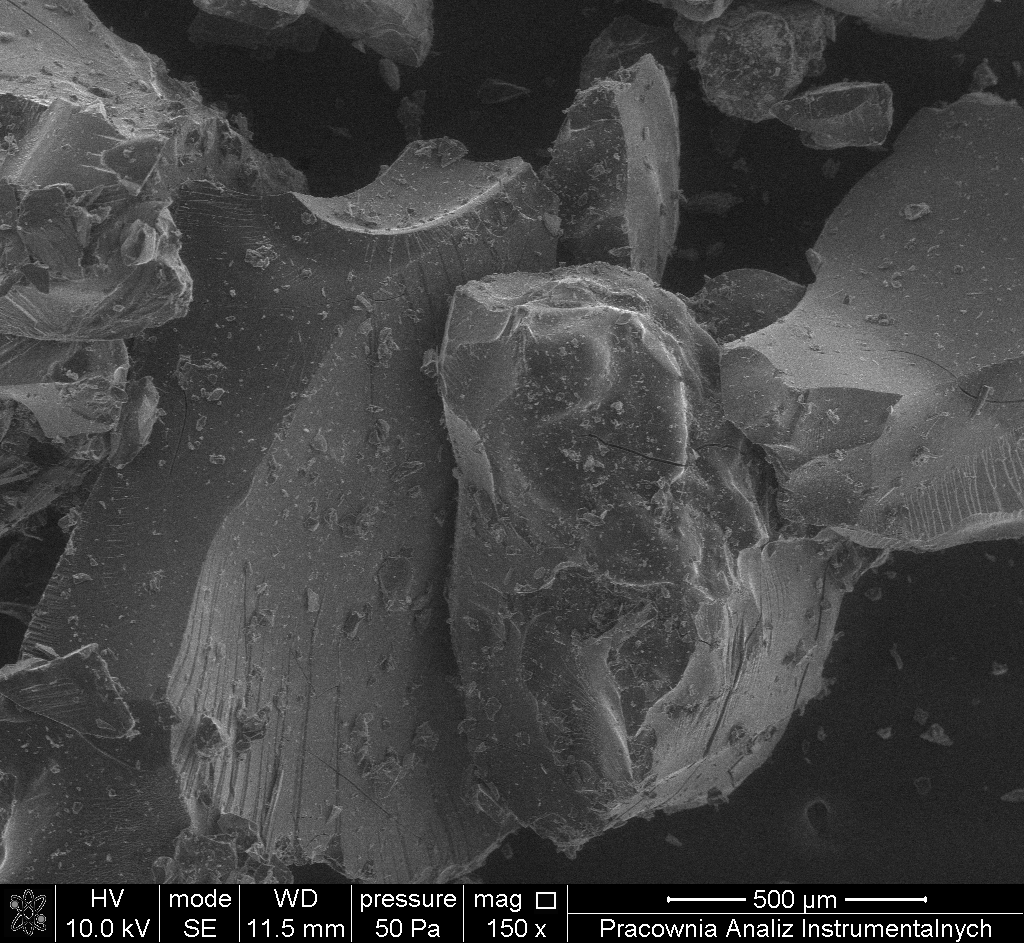


Figure S38. SEM images at 150× magnification of PVA/PS-g-P(AA-co-AM-co-AMPS).


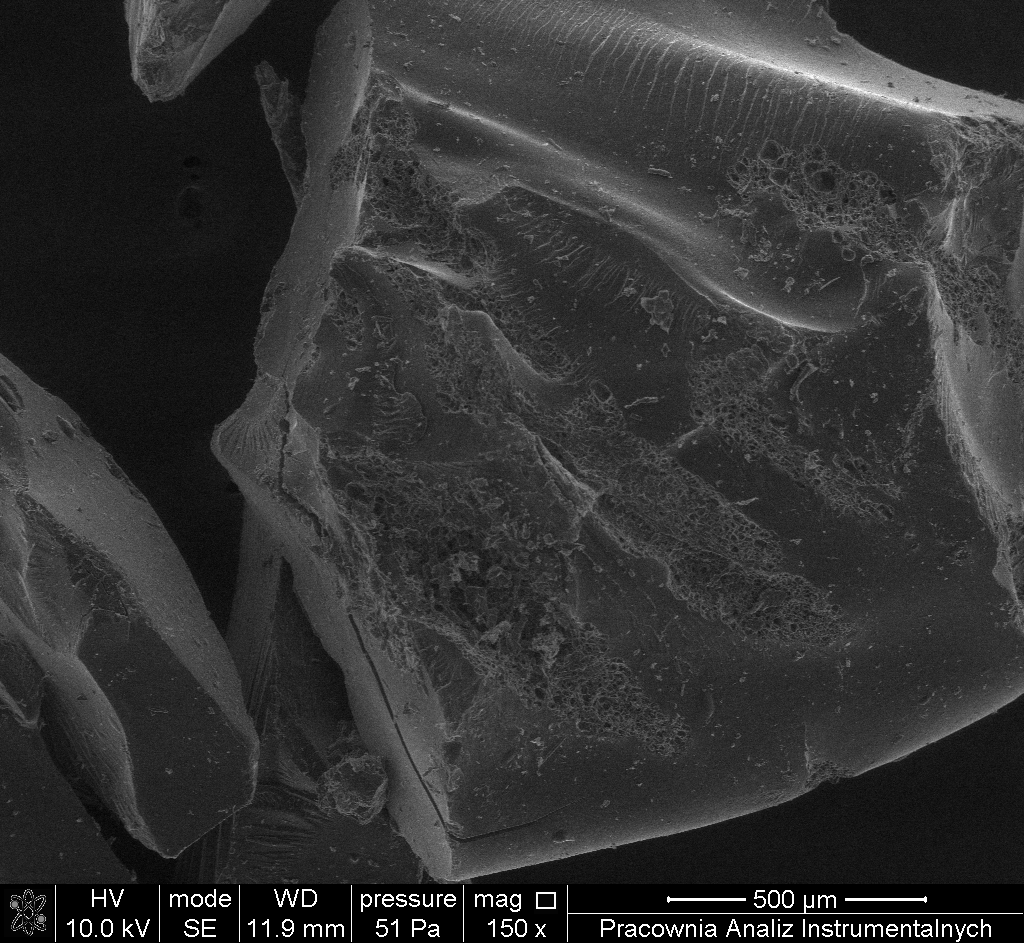
Figure S39. SEM images at 150× magnification of PVA/PS-g-P(AA-co-AM).


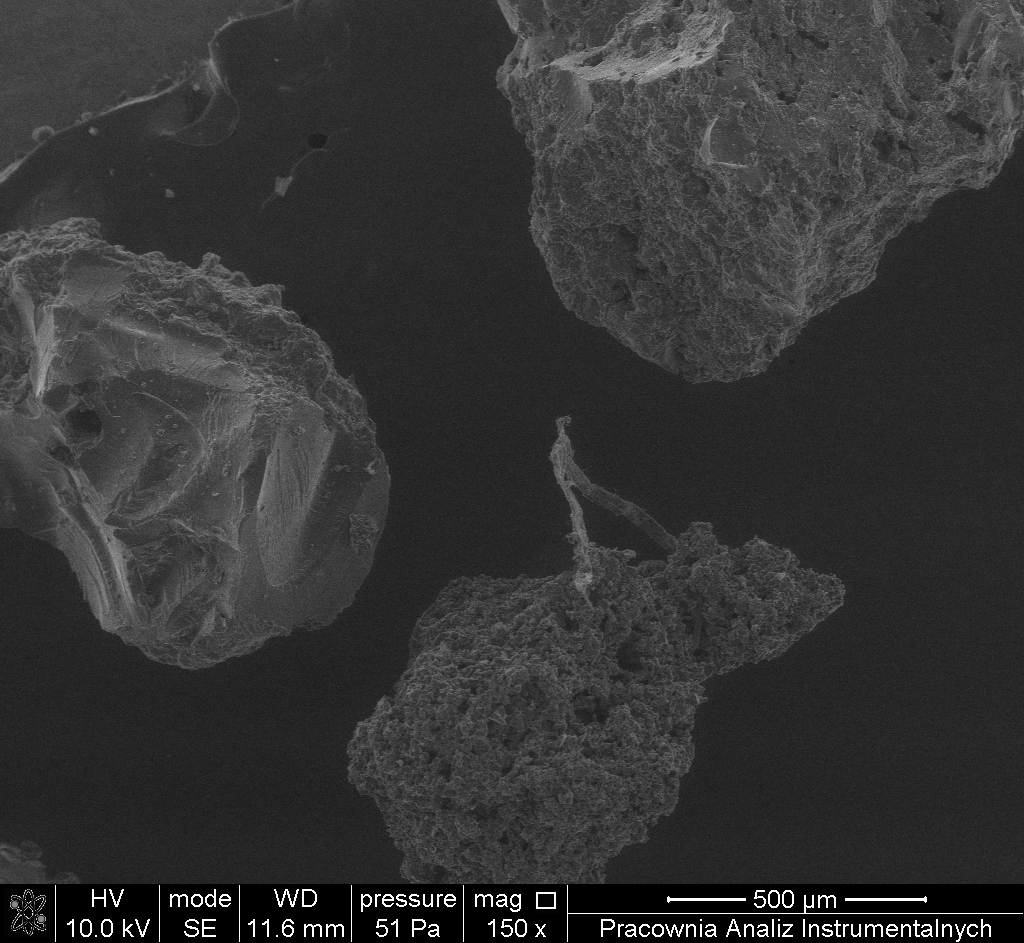


Figure S40. SEM images at 150× magnification of SS-g-P(AA-co-HEMA).


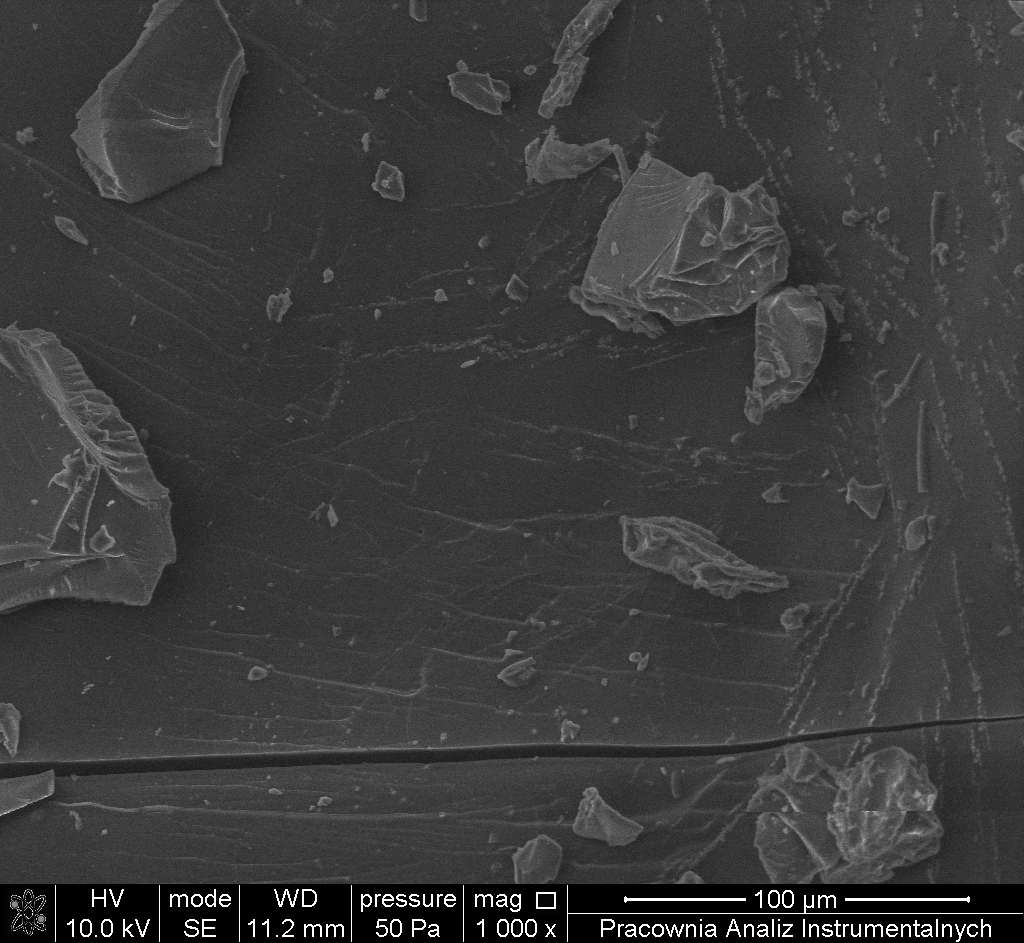


Figure S41. SEM images at 1 000× magnification of PVA/PS-g-P(AA-co-AM-co-AMPS).


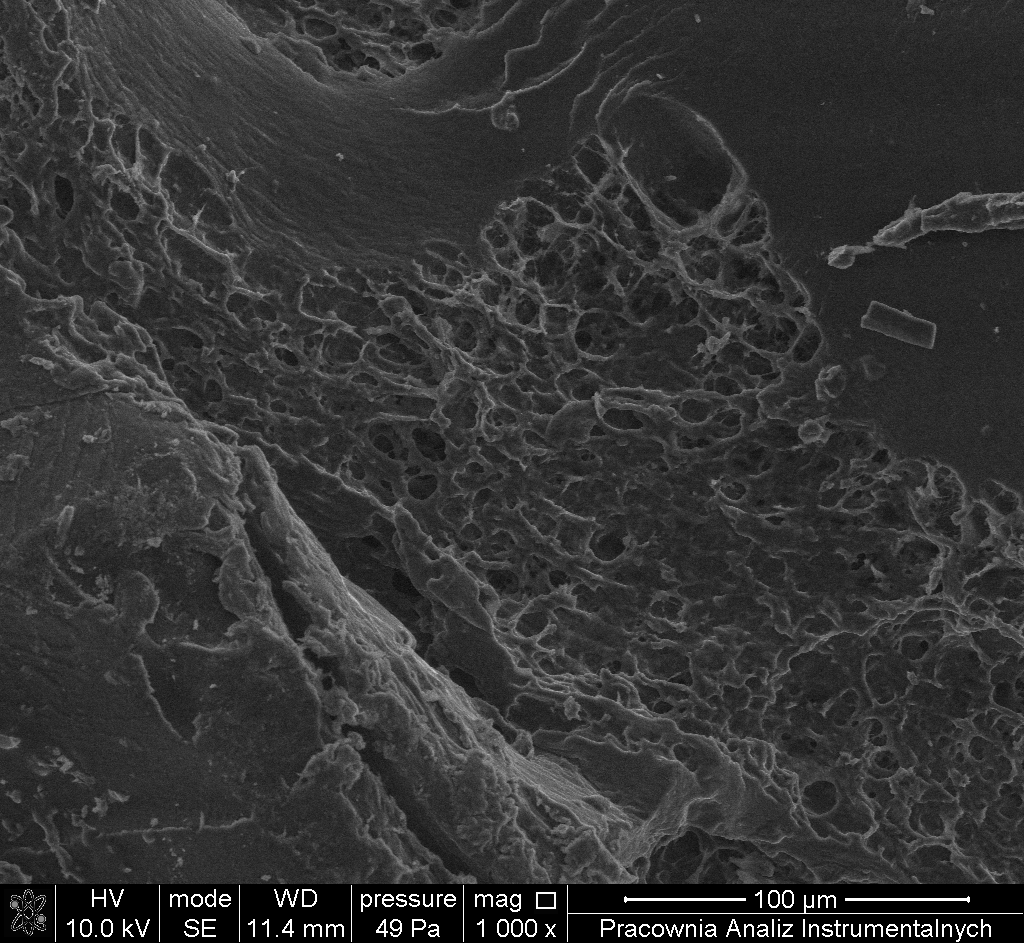


Figure S42. SEM images at 1 000× magnification of PVA/PS-g-P(AA-co-AM).


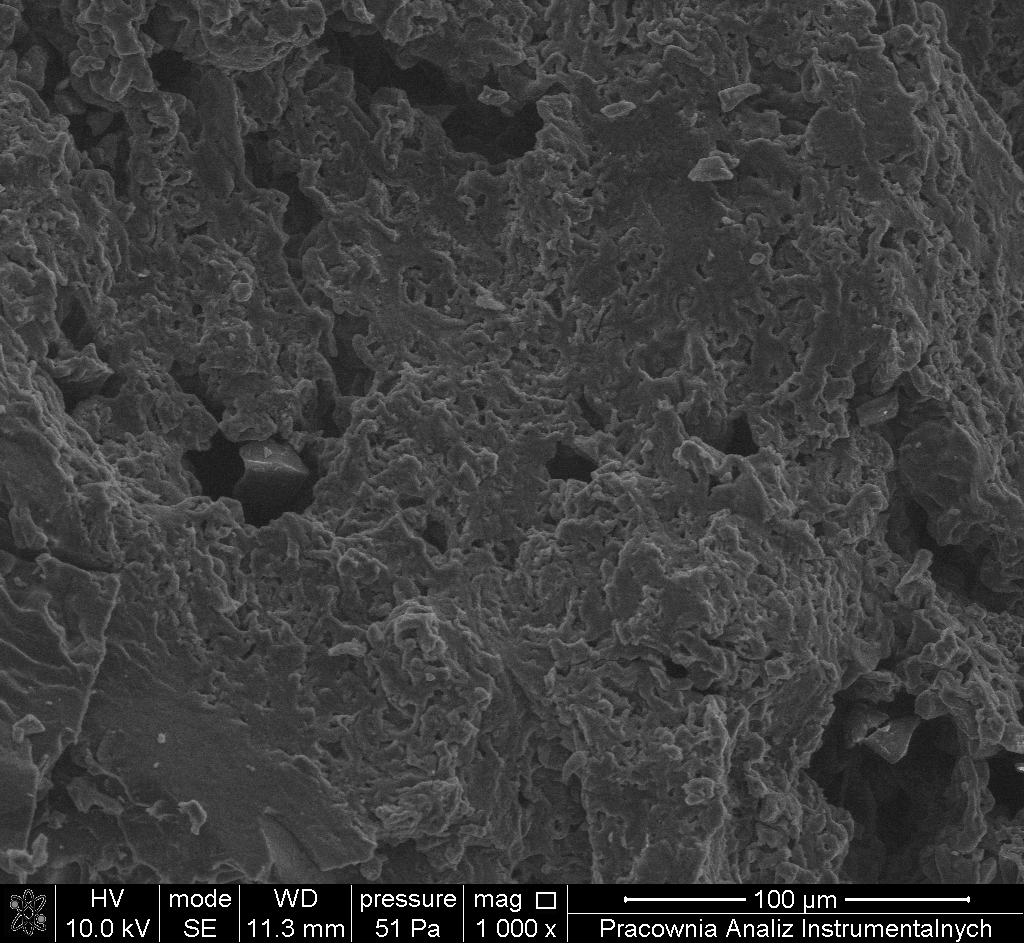


Figure S43. SEM images at 1 000× magnification of SS-g-P(AA-co-HEMA).


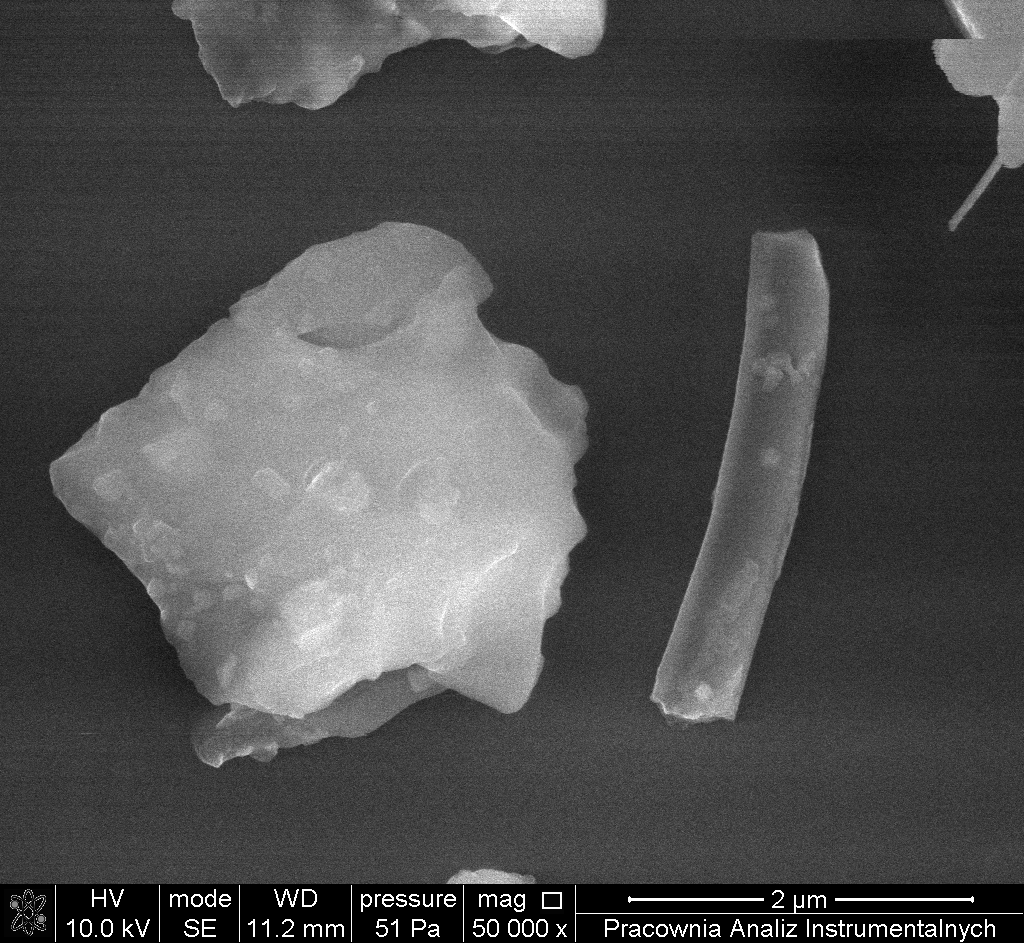


Figure 44. SEM images at 50 000× magnification of PVA/PS-g-P(AA-co-AM-co-AMPS).


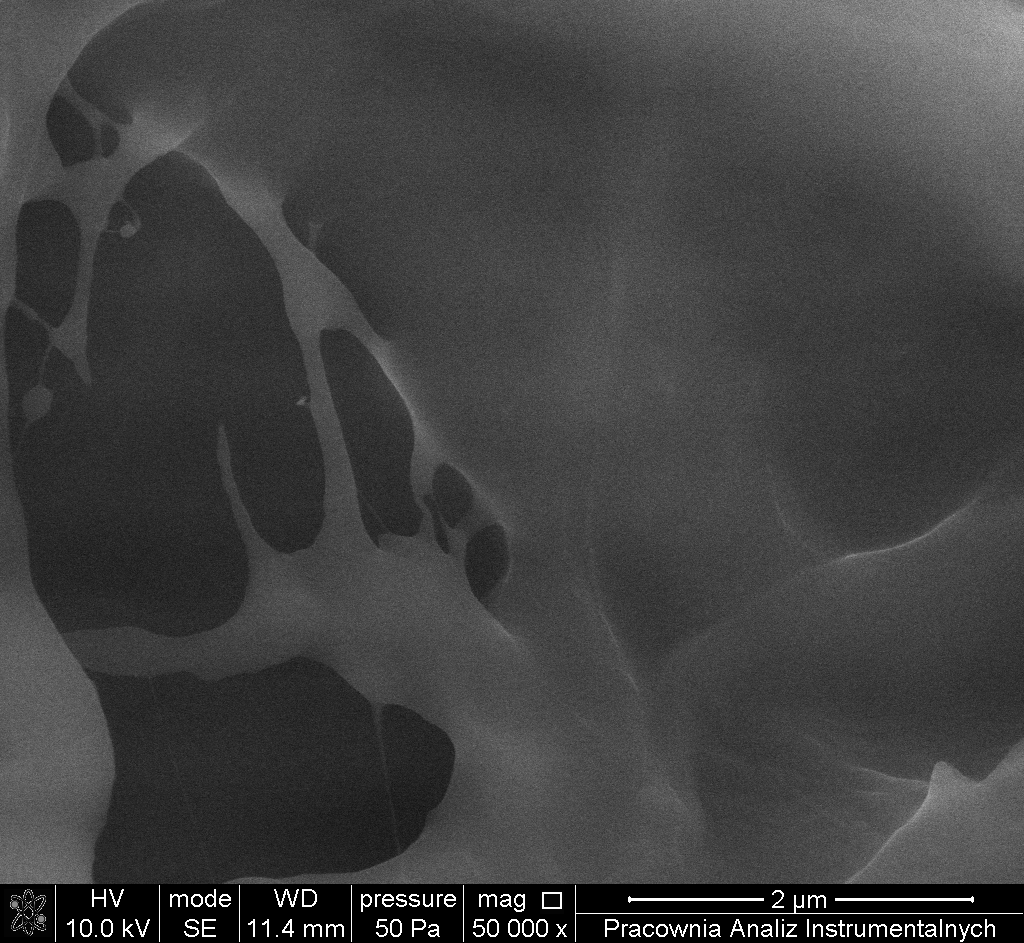


Figure S45. SEM images at 50 000× magnification of PVA/PS-g-P(AA-co-AM).


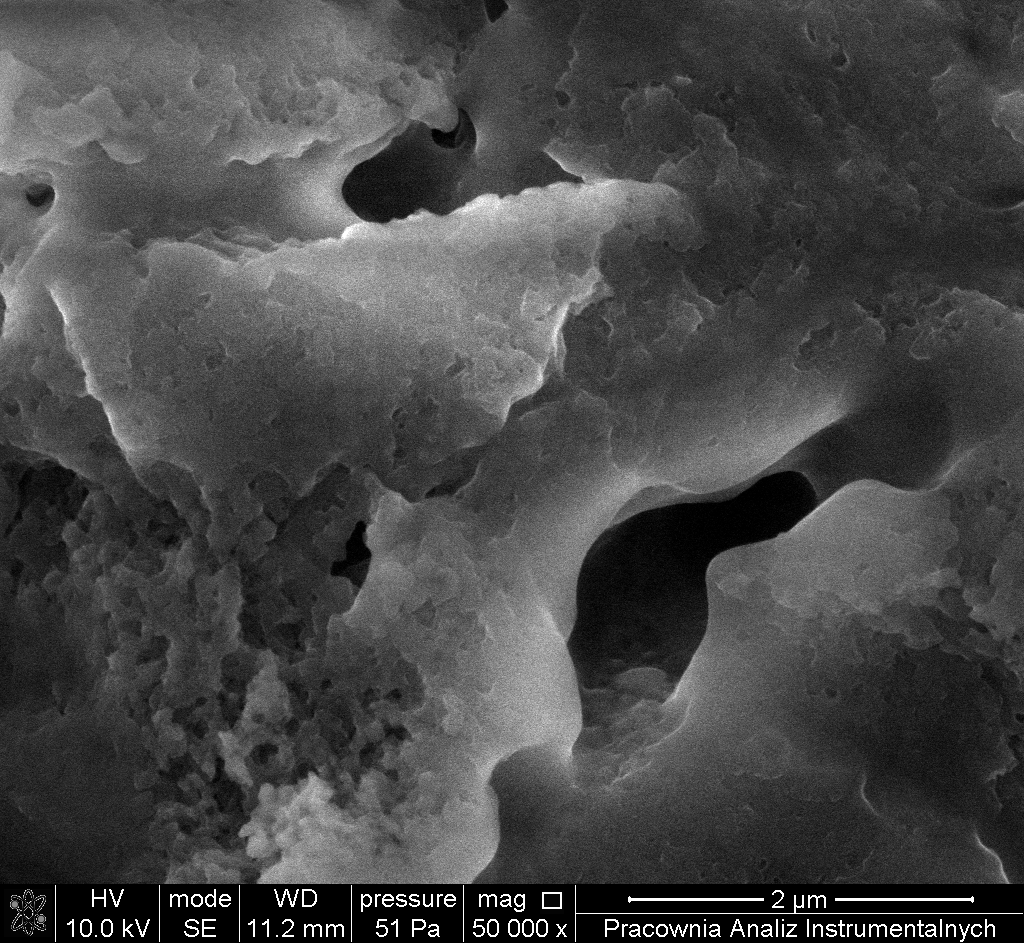


Figure S46. SEM images at 50 000× magnification of SS-g-P(AA-co-HEMA).
